# YTHDC1 phase separation drives the nuclear export of m^6^A-modified lncNONMMUT062668.2 through the transport complex SRSF3–ALYREF–XPO5 to aggravate pulmonary fibrosis

**DOI:** 10.1038/s41419-025-07608-x

**Published:** 2025-04-12

**Authors:** Shengjun Chen, Yujie Wang, Jinjin Zhang, Bo Liu, Weili Liu, Guohong Cao, Rongrong Li, Hongbo Li, Nailiang Zhai, Xiaodong Song, Songzi Zhang, Changjun Lv

**Affiliations:** 1 Department of Respiratory and Critical Care Medicine, Binzhou Medical University Hospital, Binzhou Medical University, Binzhou, China; 2 Department of Cellular and Genetic Medicine, Binzhou Medical University, Yantai, China; 3 CHA Bundang Medical Center, CHA University, Seongnam-si, Republic of Korea

**Keywords:** Long non-coding RNAs, Diagnostic markers

## Abstract

Fibroblast-to-myofibroblast differentiation is the main cytopathologic characteristic of pulmonary fibrosis. However, its underlying molecular mechanism remains poorly understood. This study elucidated that the nuclear export of lncNONMMUT062668.2 (lnc668) exacerbated pulmonary fibrosis by activating fibroblast-to-myofibroblast differentiation. Mechanistic research revealed that histone H3K9 lactylation in the promoter region of the N6-methyladenosine (m^6^A) writer METTL3 was enriched to enhance METTL3 transcription, leading to the lnc668 m^6^A modification. Meanwhile, the m^6^A reader YTHDC1 recognized m^6^A-modified lnc668 and elevated the METTL3-mediated lnc668 modification. Subsequently, phase-separating YTHDC1 promoted the nuclear export of m^6^A-modified lnc668. In this process, the phase-separating YTHDC1 formed a nuclear pore complex with serine/arginine-rich splicing factor 3, Aly/REF export factor, and exportin-5 to assist the translocation of m^6^A-modified lnc668 from nucleus to cytoplasm. After nuclear export, lnc668 facilitated the translation and stability of its host gene phosphatidylinositol-binding clathrin assembly protein to activate fibroblast-to-myofibroblast differentiation, leading to the aggravation of pulmonary fibrosis, which also depended on YTHDC1 phase separation. This study first clarified that YTHDC1 phase separation is crucial for the m^6^A modification, nuclear export, and profibrotic role of lnc668 in exacerbating pulmonary fibrosis. These findings provide new insights into the nuclear export of cytoplasmic lncRNAs and identified potential targets for pulmonary fibrosis therapy.

## Introduction

Pulmonary fibrosis is a chronic, progressive lung disease characterized by the thickening and scarring of lung tissue, leading to symptoms, such as shortness of breath, dry cough, and fatigue [1]. Epidemiologically, pulmonary fibrosis is influenced by various factors, including aging, genetic predisposition and viral infections; environmental exposure, such as exposure to silica and asbestos; and certain medications like bleomycin (BLM). Pulmonary fibrosis has an incidence of approximately 13–20 cases per 100,000 people annually, with a median survival time of 3–5 years after diagnosis [2]. Despite extensive research, the pathogenesis of pulmonary fibrosis remains poorly understood, and no effective therapies to halt disease progression. Fibroblasts are responsible for maintaining normal tissue structure and repairing damage in the lungs. Abnormal repair is accompanied with fibroblast activation and differentiation into myofibroblasts [3]. Myofibroblasts synthesize and secrete extracellular matrix components, such as collagen, various cytokines, and growth factors, such as transforming growth factor (TGF)-β1, through autocrine and paracrine mechanisms. These phenomena further promote the activation and differentiation of fibroblasts, thus exacerbating fibrosis [4, 5]. Therefore, studying the differentiation of fibroblasts into myofibroblasts is crucial for understanding and potentially treating pulmonary fibrosis.

Long noncoding RNAs (lncRNAs) are RNA transcripts, typically exceeding 200 nucleotides in length, that cannot encode proteins. On the basis of their genomic origin or function, lncRNAs are classified into enhancer lncRNAs, promoter lncRNAs, antisense lncRNAs, mitochondrial DNA–encoded lncRNAs, circular lncRNAs, and snoRNA-derived lncRNAs with snoRNA ends [6]. These lncRNAs regulate nearly all kinds of cell biological processes, such as cell differentiation, proliferation, migration, and death, by participating in chromatin remodeling, DNA replication, and RNA transcription, translation, and modification [7–12]. Research has revealed that the binding of ATF3 to the promoter region of lncIAPF enhances lncIAPF transcription to inhibit autophagy by promoting the expression of the negative autophagy regulatory factors EZH2, STAT1, and FOXK1. This process accelerates the differentiation of fibroblasts into myofibroblasts, leading to the development of pulmonary fibrosis [13, 14]. lncITPF can promote the development of pulmonary fibrosis by targeting hnRNP-L, which depends on the expression of its host gene ITGBL1 [15]. The lncPFAR promotes lung fibroblast activation and ECM deposition by competitively binding miR-138 to regulate the YAP1–Twist axis, leading to the development of idiopathic pulmonary fibrosis (IPF) [16]. Lnc00673 inhibits miR-150-5p through a sponge mechanism, thereby indirectly upregulating the expression of genes involved in cell proliferation, migration, and invasion [17]. Multiple studies have demonstrated that these cytoplasmic lncRNAs are involved in lung diseases. However, the nuclear export of these cytoplasmic lncRNAs remains poorly characterized.

This present work on the lncRNA NONMMUT062668.2 (lnc668) is a follow-up to our previous research on differentially expressed lncRNAs during pulmonary fibrogenesis [18]. lnc668, with a full length of 1139 bp, is located on mouse chromosome 7 and is highly homologous to the human genome. It is transcribed from the complementary strand of its host gene phosphatidylinositol-binding clathrin assembly protein (PICALM) located on human chromosome 11 (11q14.2) [19] and is an antisense lncRNA. In our research on lnc668, we found that the nuclear export of lnc668 depends on YTHDC1 liquid–liquid phase separation (LLPS). Phase separation, as a regulatory pattern of intracellular molecules, can organize and segregate different biomolecules within cells by forming dynamic, reversible droplets or condensates. These membraneless, high-concentration liquid condensates or punctate structures can be influenced by factors, such as temperature, pH, macromolecules, and solute concentrations [20–22]. Under normal conditions, these phase separation structures coexist in a dynamically reversible manner. When they are stimulated by external factors, they tend to stabilize into high-density gel-like phases [23–25], which can affect various cellular functions. CTCF organizes inter-A compartment interactions through RYBP-dependent phase separation to regulate chromatin remodeling and gene expression [26]. The properties of TDP-43 phase separation specify its RNA-binding and regulatory repertoire in neurodegenerative diseases [27]. SGF29 undergoes phase separation to reinforce cellular aging [28]. In FUS-associated neurodegeneration, the phase separation of FUS is suppressed by the nuclear import receptor Transportin and arginine methylation [29]. However, the role of phase separation has not yet been deeply explored in pulmonary fibrosis research. In this study, we focused on how phase-separating YTHDC1 promotes the nuclear export of lnc668 to accelerate fibroblast-to-myofibroblast differentiation, resulting in pulmonary fibrosis. Our work can provide an important experimental, fundamental, and theoretical strategy to develop new treatments for pulmonary fibrosis.

## Results

### Highly expressed lnc668 promoted fibroblast-to-myofibroblast differentiation during pulmonary fibrogenesis

In our previous study [18], RNA sequencing revealed that in mice with BLM-induced pulmonary fibrosis, the RNA transcript of NONMMUT062668.2 was significantly highly expressed compared with other transcripts (Supplementary Fig. 1a). UCSC Genome Browser (http://genome.ucsc.edu) demonstrated that NONMMUT062668.2 is located on chromosome 7 of the mouse genome and transcribed from its host gene PICALM. The open reading frame finder from NCBI showed that NONMMUT062668.2 has no valid Kozak sequence. Coding Potential Assignment Tool (http://lilab.research.bcm.edu/cpat/index.php), Coding Potential Calculator (http://cpc.cbi.pku.edu.cn/), and Coding–NonCoding Index Tool (https://github.com/www-bioinfo-org/CNCI) illustrated that NONMMUT062668.2 lacks protein-coding capability. Hence, NONMMUT062668.2 was selected for further study and is abbreviated as lnc668 in this text.

**Fig. 1:**
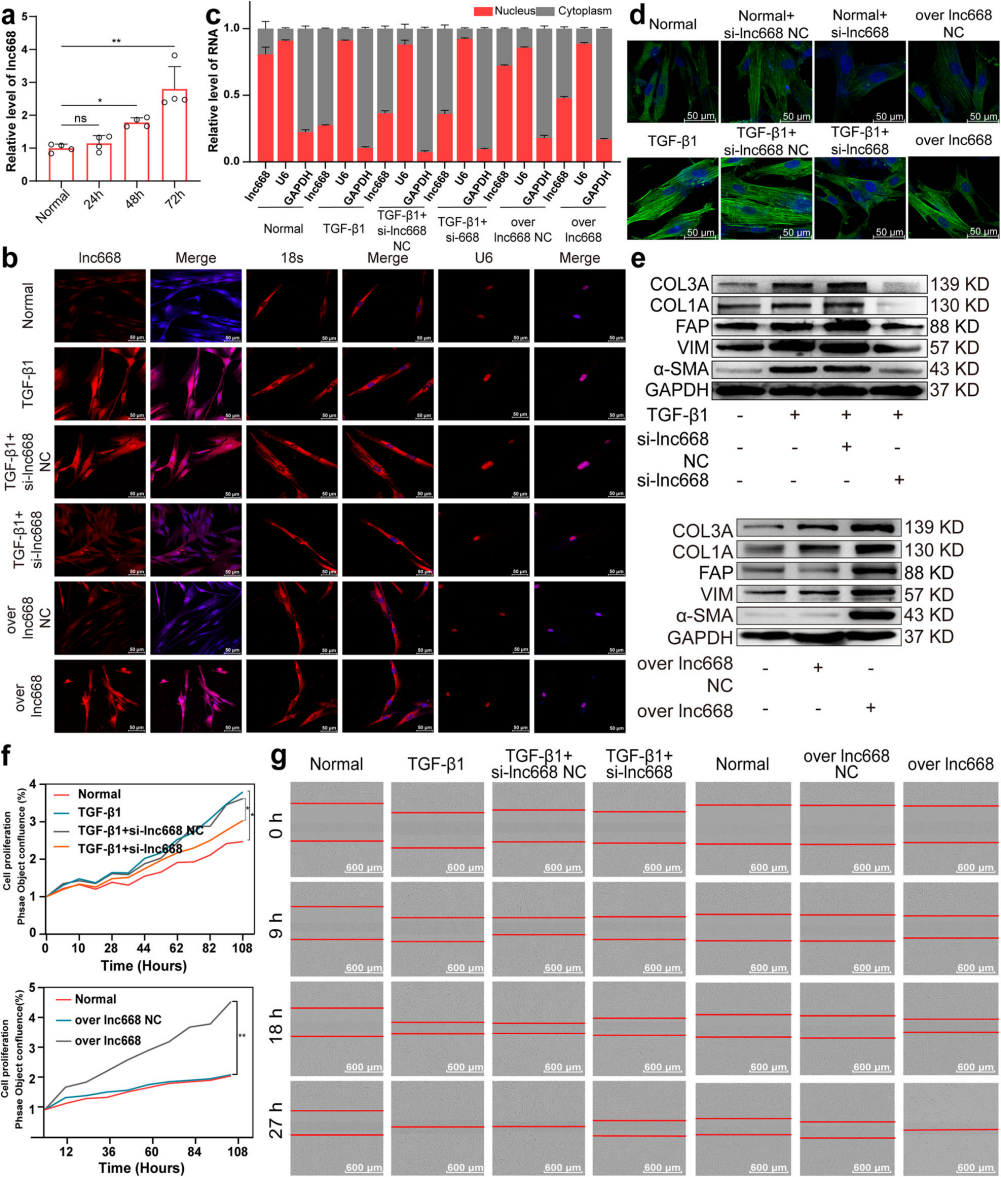
lnc668 promoted fibroblast-to-myofibroblast differentiation in TGF-β1-activated MRC-5 cells. **a** qRT-PCR confirmed that the expression of lnc668 gradually increased in the TGF-β1 group compared with that in the control group and peaked at 72 h. **b** RNA FISH images revealed that lnc668 was present in the nucleus and cytoplasm but was predominantly present in the nucleus. After TGF-β1 stimulation, lnc668 was upregulated in the nucleus and cytoplasm, with a more pronounced increase in the cytoplasm than in the nucleus. After si-lnc668 treatment, lnc668 levels were downregulated in the nucleus and cytoplasm, whereas lnc668 overexpression resulted in upregulation in both compartments, with a more significant increase in the nucleus than in the cytoplasm. **c** The nuclear-cytoplasmic separation experiments confirmed that lnc668 was primarily expressed in the nucleus of MRC-5 cells, with TGF-β1 stimulation increasing its cytoplasmic levels. Overexpression of lnc668 similarly elevated its cytoplasmic expression, while lnc668 interference disrupted its nuclear-cytoplasmic shuttling. **d** Immunofluorescence images revealed that cells treated with TGF-β1 exhibited a spindle shape and an increased expression of the α-SMA protein compared with the normal cell group. Comparison with the TGF-β1 group revealed that α-SMA expression was inhibited by lnc668 silencing and promoted by lnc668 overexpression. Blue represents nuclei marked with DAPI. Green indicates the presence of α-SMA in the cytoplasm. **e** Western blot analysis revealed that lnc668 intervention downregulated the expression levels of FAP, α-SMA, VIM, COL1A, and COL3A, whereas lnc668 overexpression led to increased levels of these proteins. **f** IncuCyteS3 analysis revealed that cell proliferation decreased in the si-lnc668 group and increased in the lnc668 overexpression group. **g** Scratch assay results showed that cell migration in the si-lnc668 group decreased and that in the lnc668 overexpressing group increased compared with that in the control group.

A homology comparison revealed that mouse lnc668 shares a highly homologous sequence with humans. The full-length human lnc668 was obtained through RACE and PCR amplification, and agarose gel electrophoresis confirmed that lnc668 was indeed present (Supplementary Fig. 1b–d). Subsequently, human embryonic lung fibroblasts (MRC-5 cells) were activated with 5 ng/mL TGF-β1 to establish a cell differentiation model of pulmonary fibrosis. Quantitative reverse transcription polymerase chain reaction (qRT-PCR) verified that lnc668 expression in the TGF-β1 group gradually increased relative to that in the normal group, and the highest expression was observed at 72 h (Fig. 1a). A lnc668 smart silencer (si-lnc668) and lnc668 overexpression plasmids were designed on the basis of the full-length sequence of lnc668. After confirming their efficiency (Supplementary Fig. 1d), gain- and loss-function studies were performed to detect the role of human lnc668 during pulmonary fibrogenesis. The subcellular localization of lnc668 was first determined through RNA fluorescence in situ hybridization (RNA FISH) experiments (Fig. 1b). Images demonstrated that lnc668 was present in the nucleus and cytoplasm of MRC-5 cells, showing higher expression in the nucleus than in the cytoplasm. After TGF-β1 stimulation, the expression of lnc668 was upregulated in the nucleus and cytoplasm, exhibiting a more significant increase in the cytoplasm in the treatment group than in the normal group, suggesting that lnc668 may be shuttled from the nucleus to the cytoplasm under TGF-β1 stimulation. The expression of lnc668 decreased in the nucleus and cytoplasm to the same extent after interference, indicating that si-lnc668 cannot cause lnc668 nucleocytoplasmic shuttling. After overexpression, lnc668 was upregulated in the nucleus and cytoplasm, presenting a pronounced increase in the cytoplasm similar to that shown by TGF-β1. The nuclear-cytoplasmic separation experiments further confirmed that lnc668 was expressed in both the nucleus and cytoplasm of MRC-5 cells, with higher expression levels in the nucleus compared to the cytoplasm. After TGF-β1 stimulation, the expression of lnc668 in the cytoplasm significantly increased, more prominently than in the control group. Following lnc668 interference, the expression of lnc668 in the cytoplasm was higher than in the nucleus, indicating that si-lnc668 did not promote the nuclear-cytoplasmic shuttling of lnc668. In contrast, overexpression of lnc668 led to a significant increase in its cytoplasmic levels, similar to the TGF-β1 treatment group (Fig. 1c). Immunofluorescence images depicted that compared with the normal group cells, MRC-5 cells treated with TGF-β1 appeared spindle-shaped and exhibited an increased expression of alpha smooth muscle actin (α-SMA), a key indicator of myofibroblasts. Compared with TGF-β1, the lnc668 smart silencer suppressed the expression of α-SMA (Fig. 1d). The overexpression of lnc668 significantly increased the expression of α-SMA. Western blot analysis demonstrated that TGF-β1 increased the expression levels of the differentiation-related protein fibroblast activation protein α (FAPα) and the fibrosis markers collagen type I alpha (COL1A), collagen type III alpha (COL3A), and vimentin (VIM). The expression levels of these proteins decreased following treatment with si-lnc668 and increased under lnc668 overexpression (Fig. 1e). Proliferation and migration enhanced under TGF-β1 stimulation but were weakened by si-lnc668. Similar to TGF-β1, lnc668 overexpression enhanced proliferation and migration (Fig. 1f, g). The above data indicated that lnc668 upregulation promotes fibroblast-to-myofibroblast differentiation in vitro.

### YTHDC1 assisted METTL3 to promote the N6-methyladenosine modification of lnc668

**Fig. 2:**
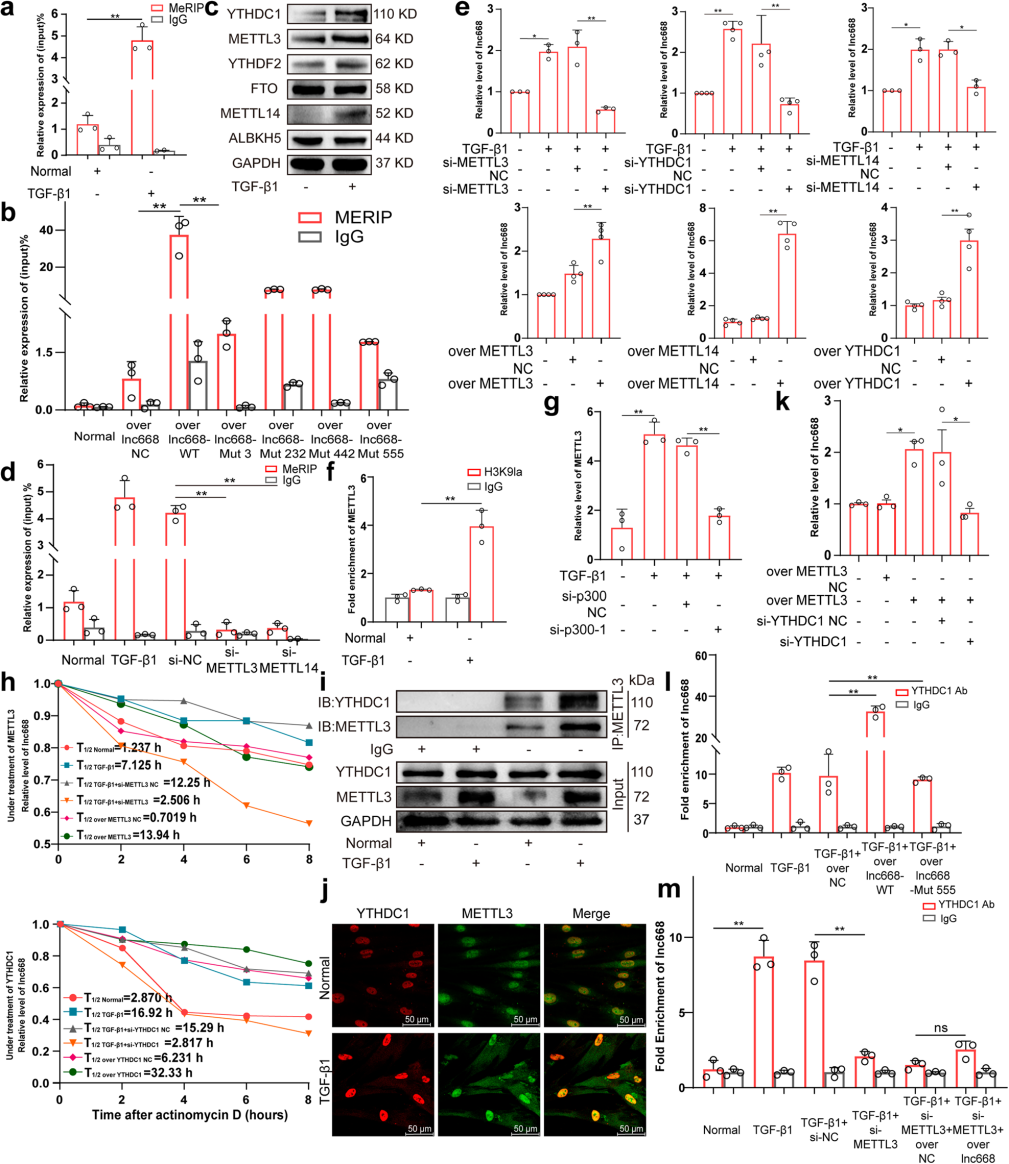
YTHDC1 recognized the m^6^A modification of lnc668 to promote the METTL3-mediated m^6^A modification of lnc668. **a** MeRIP-qPCR confirmed the presence of m^6^A modification in lnc668, along with increased methylation after TGF-β1 stimulation. The IgG group served as a negative control to exclude any background signal from nonspecific binding to the antibody. **b** MeRIP-qPCR confirmed that the methylation level in the over lnc668-WT group significantly increased, whereas that in the over lnc668-Mut 3 groups decreased. The mutation at position 555 led to a significant reduction in m^6^A methylation in a METTL3-dependent manner. **c** Western blot analysis confirmed that in the TGF-β1 group, the expression levels of METTL3, METTL14, YTHDC1, and YTHDF2 increased, whereas those of FTO and ALBKH5 decreased. **d** MeRIP experiments revealed that si-METTL3 and si-METTL14 reduced the m^6^A modification of lnc668. **e** qRT-PCR results verified that METTL3, METTL14, and YTHDC1 overexpression increased lnc668 expression, whereas si-METTL3, si-METTL14, and si-YTHDC1 reduced lnc668 expression. **f** ChIP-PCR results revealed that histone H3K9la was highly enriched in the promoter region of METTL3 in the TGF-β1 group. **g** qRT-PCR revealed that compared with TGF-β1 stimulation, H3K9la inhibition by si-p300 diminished METTL3 expression. **h** qRT-PCR results illustrated that lnc668 stability significantly increased in the METTL3 and YTHDC1 overexpression group but significantly decreased in the interference group. **i** Co-IP experiments enriched the YTHDC1 protein, and Western blot analysis discovered high expression levels of YTHDC1 and METTL3 in the TGF-β1 group. Their interaction was observed in the IP group. **j** Immunofluorescence exhibited that the colocalization of YTHDC1 and METTL3 increased in the nuclei. **k** Rescue experiments confirmed that METTL3 overexpression increased lnc668 expression. Interference with YTHDC1 reduced lnc668 expression and reversed the effect of METTL3 on lnc668 expression. **l** RIP-qPCR experiments indicated that the binding of YTHDC1 with lnc668 increased after TGF-β1 stimulation. The amount of bound lnc668 in the lnc668 overexpression-WT group was greater than that in the TGF-β1 stimulation group. The enrichment of lnc668 significantly reduced in the lnc668 overexpression-Mut group. **m** RIP experiments showed that METTL3 interference significantly reduced YTHDC1 binding to lnc668, even with lnc668 overexpression.

Given that N6-methyladenosine (m^6^A) is the most prevalent RNA modification and participates in lncRNA biogenesis to control the development of various diseases [30, 31], the existence and specific role of m^6^A in lnc668 regulation were further explored. In accordance with SRAMP predictions (https://www.cuilab.cn/sramp), we obtained three highly consistently methylated sites (A232, 442, and 555) and two lowly consistently methylated sites (A43 and 476) with RRACH motifs in the lnc668 sequence (Supplementary Fig. 2a). Primers were designed for the sites of A232, 442, and 555. Methylated RNA immunoprecipitation (MeRIP) showed that m^6^A modification was present in lnc668 and methylation was more pronounced in TGF-β1-stimulated MRC-5 cells than in normal cells (Fig. 2a). We constructed overexpression vectors for wild-type (lnc668-WT) and mutant lnc668, wherein the adenine residues at positions 232, 442, and 555 (lnc668-Mut 3) were substituted by cytosine (A-C mut). Subsequently, single mutations were introduced at each adenine residue of lnc668 (lnc668-Mut 232, lnc668-Mut 442, and lnc668-Mut 555), and these vectors were transfected into MRC-5 cells to assess whether these m^6^A residues are essential for m^6^A modification of lnc668. We found that compared with those in wild-type transcripts, m^6^A levels had significantly decreased in lnc668 mutant transcripts. These results indicated that positions 232, 442, and 555 are predominantly responsible for lnc668 m^6^A modification, with a notable reduction in m^6^A methylation observed only when the mutation occurred at position 555. This finding indicates that position 555 is a critical site for m^6^A modification of lnc668 (Fig. 2b). Western blot analysis revealed that after TGF-β1 stimulation, the m^6^A writers METTL3 and METTL14 and the readers YTHDC1 and YTHDF2 significantly increased, whereas the expression of the erasers FTO and ALBKH5 decreased only weakly (Fig. 2c). Therefore, we further illustrated whether METTL3/METTL14 directly promotes the m^6^A modification of lnc668 following TGF-β1 stimulation. MeRIP illustrated that interference with METTL3 and METTL14 directly reduced the m^6^A modification of lnc668, indicating that METTL3 and METTL14 can directly promote the m^6^A modification of lnc668 (Fig. 2d). METTL3 can directly catalyze methylation reactions using *S*-adenosylmethionine as the methyl donor. METTL14, although lacking direct catalytic activity, enhances METTL3’s catalytic efficiency and specificity by forming a heterodimer complex with METTL3 [22]. YTHDC1 is a key m^6^A reader protein, with its conserved YTH domain specifically recognizing and binding to m^6^A-modified RNA. Next, we designed gain- and loss-of-function studies to explore the effect of the above three molecules on lnc668. qRT-PCR revealed that METTL3, METTL14, and YTHDC1 overexpression resulted in increased lnc668 expression, whereas si-METTL3, si-METTL14, and si-YTHDC1 decreased lnc668 expression (Fig. 2e; Supplementary Fig. 2b–d). Given that METTL3 can directly catalyze methylation and METTL14 plays an auxiliary role, METTL3 was subsequently selected as the focus of this study.

Pathologic myofibroblast phenotypes are related to the secretion of lactate and other glycolytic intermediates in glycolysis. As a result, histone lactylation participates in disease generation and development [32]. However, the regulation of histone lactylation in pulmonary fibrosis is still unknown. We found that H3K9la was significantly elevated in TGF-β1-stimulated MRC-5 cells (Supplementary Fig. 2e). Furthermore, chromatin immunoprecipitation (ChIP) was conducted to explore the regulatory effect of histone lactylation on METTL3 transcription (Fig. 2f). We discovered that TGF-β1 significantly upregulated the enrichment of H3K9la at the METTL3 promoter site and the inhibition of H3K9la levels by using si-p300 downregulated the mRNA level of METTL3 (Fig. 2g, Supplementary Fig. 2f, g). This finding indicated that H3K9la can promote METTL3 transcription.

m^6^A modification plays important roles in the biogenesis, stability, nuclear export, splicing, and translation of RNAs. The half-life experiment validated that the stability of lnc668 was weakened by si-METTL3 and si-YTHDC1 and enhanced by METTL3 and YTHDC1 overexpression (Fig. 2h). Co-immunoprecipitation (Co-IP) results elucidated that the binding between YTHDC1 and METTL3 enhanced under TGF-β1 stimulation (Fig. 2i). Immunofluorescence images revealed that YTHDC1 and METTL3 colocalized in the nucleus and were upregulated in the TGF-β1 group relative to in the control group (Fig. 2j). Our rescue experiment results further proved that the expression of lnc668 was elevated in the METTL3 overexpression group relative to that in the normal group. Interference with YTHDC1 reduced lnc668 expression and reversed the increase caused by METTL3 overexpression (Fig. 2k). Subsequently, enriching the YTHDC1 protein through RIP-qPCR showed that the binding interaction between YTHDC1 and lnc668 increased in the TGF-β1 stimulation group compared with that in the normal group. Compared with TGF-β1 stimulation alone, the further overexpression of wild-type lnc668-WT on top of TGF-β1 stimulation increased the interaction. Meanwhile, the lnc668 mutant transcripts (lnc668-Mut 555) showed significantly lessened interaction with YTHDC1 (Fig. 2l). The RNA immunoprecipitation (RIP) results showed that, compared to the TGF-β1 stimulation group, interference with METTL3 significantly reduced the binding of YTHDC1 to lnc668. Even with the overexpression of lnc668, the binding of YTHDC1 to lnc668 did not increase (Fig. 2m). The above findings suggested that YTHDC1 can recognize m^6^A-modified lnc668 and promote the METTL3-mediated methylation of lnc668.

### Nuclear export of m^6^A-modified lnc668 depended on YTHDC1 phase separation

**Fig. 3:**
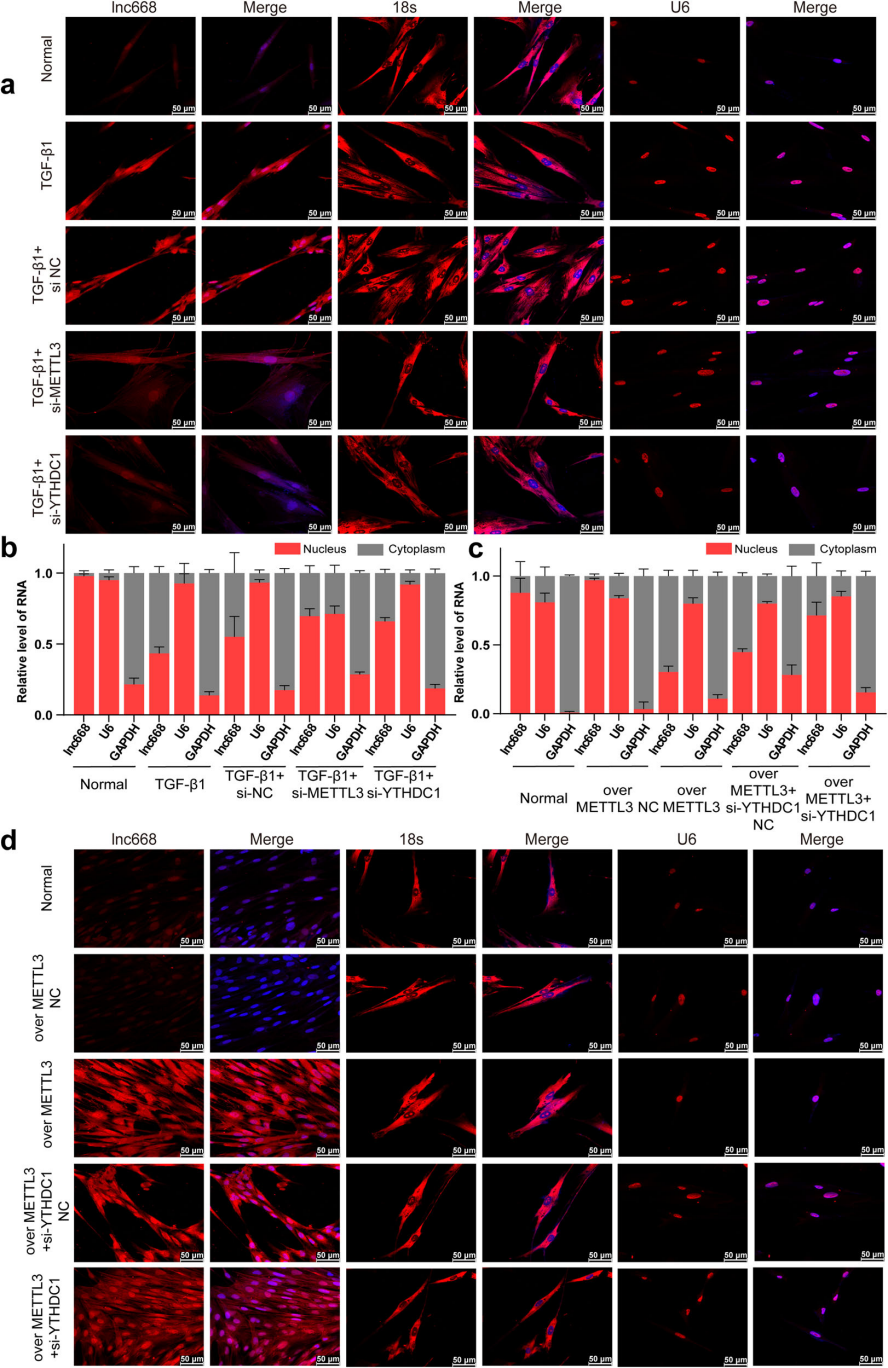
YTHDC1 promoted the nuclear export of lnc668 by facilitating METTL3-mediated m^6^A modification. **a** RNA-FISH experiments were conducted to measure the expression levels and nuclear-cytoplasmic distribution of lnc668 in MRC-5 cells. lnc668 primarily localized in the nuclei, with minimal expression in the cytoplasm. Under the action of TGF-β1, the expression of lnc668 increased in the nucleus and cytoplasm. However, its increase in the nucleus was less pronounced than that in the cytoplasm. Silencing METTL3 and YTHDC1 led to a reduction in the expression of lnc668 in the nucleus and cytoplasm, with a more pronounced decrease in the cytoplasm than in the nucleus. DAPI (blue) was used to stain nuclei. **b** Nuclear–cytoplasmic separation experiments revealed that in the normal group, lnc668 was primarily expressed in the nucleus. After TGF-β1 stimulation, lnc668 expression decreased in the nucleus and increased in the cytoplasm. Interference with METTL3 and YTHDC1 decreased cytoplasmic lnc668 expression and increased nuclear lnc668 expression. **c** Nuclear–cytoplasmic fractionation rescue experiments showed that in the METTL3 overexpression group, the expression of lnc668 decreased in the nucleus and increased in the cytoplasm. si-YTHDC1 reduced lnc668 expression in the cytoplasm and increased that in the nucleus, which reversed the effect of METTL3 overexpression on lnc668 in the cytoplasm and nucleus. **d** RNA-FISH experiments demonstrated that the lnc668 expression increased in the nucleus and cytoplasm under METTL3 overexpression, with a more pronounced increase in the cytoplasm than in the nucleus. After YTHDC1 was silenced, lnc668 expression decreased in the cytoplasm and increased in the nucleus.

Next, we studied the nuclear export of m^6^A-modified lnc668. RNA-FISH images depicted that lnc668 expression increased in the nucleus and cytoplasm, with a more significant increase in the cytoplasm in the TGF-β1 group than in the normal group. However, interference with METTL3 and YTHDC1 led to a reduction in lnc668 expression in both compartments, with a more pronounced decrease in the cytoplasm than in the nucleus (Fig. 3a). Nuclear–cytoplasmic separation experiments were performed to confirm this point further (Fig. 3b). The results demonstrated that in the normal group, lnc668 was mainly expressed in the nucleus. Under TGF-β1 stimulation, although lnc668 was expressed in the nucleus and cytoplasm, its expression decreased in the nucleus and increased in the cytoplasm. This condition was consistent with the phenomenon observed in RNA-FISH experiments. After interference with METTL3 and YTHDC1, the expression of lnc668 in the cytoplasm decreased, whereas that in the nucleus increased. The rescue experiment on the nuclear export of lnc668 confirmed that the expression of lnc668 increased in the cytoplasm and decreased in the nucleus under the action of METTL3 overexpression. Upon the silencing of YTHDC1 expression, cytoplasmic lnc668 expression decreased, and nuclear lnc668 expression increased, which reversed the trend caused by METTL3 overexpression (Fig. 3c). Compared with that in the normal group, the expression of lnc668 in the nucleus and cytoplasm increased under METTL3 overexpression, with a more pronounced increase in the cytoplasm than in the nucleus. After YTHDC1 expression was silenced, the expression of lnc668 decreased in the cytoplasm and increased in the nucleus, reversing the trend caused by METTL3 overexpression (Fig. 3d). The above data indicated that the nuclear export of m^6^A-modified lnc668 depends on YTHDC1.

**Fig. 4:**
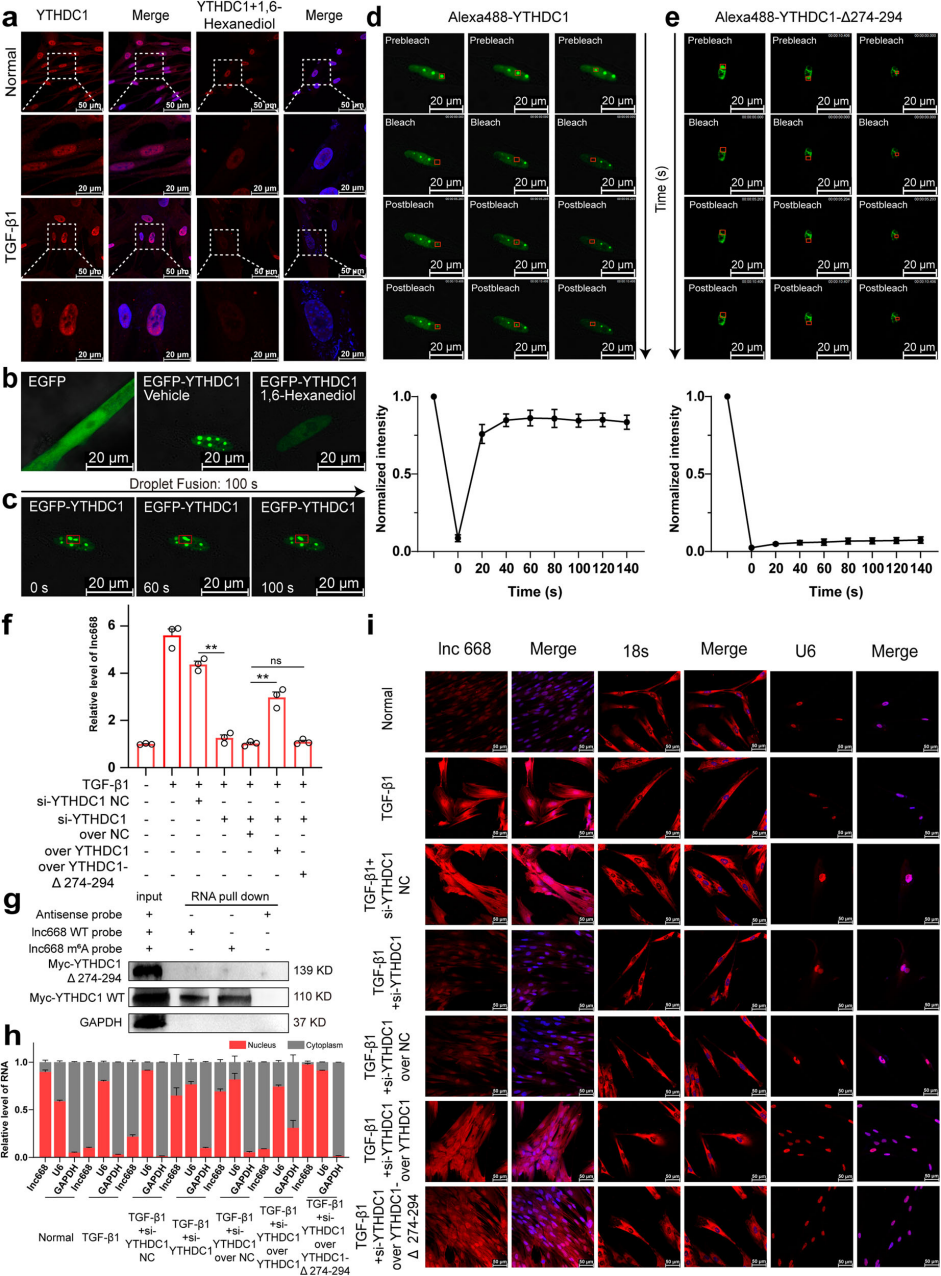
Nuclear export of lnc668 depended on YTHDC1 phase separation. **a** Immunofluorescence demonstrated that treatment with the phase separation inhibitor 1,6-hexanediol resulted in a reduction in nuclear condensates in the TGF-β1 and normal groups. **b** The EGFP group did not exhibit nuclear punctate structures. By contrast, cells overexpressing EGFP-YTHDC1 displayed numerous visible nuclear punctate structures. After 1,6-hexanediol addition, the nuclear punctate structures significantly reduced. **c** Time-lapse live-cell imaging demonstrated that EGFP-YTHDC1 nuclear droplets exhibited rapid phase separation dynamics. Two adjacent droplets fused within 60 s. **d** FRAP experiments revealed rapid fluorescence recovery in a photobleached region of a nuclear punctate structure in the overexpressed EGFP-YTHDC1 group. The fluorescence intensity in the bleached area recovered to 50% of the initial intensity within 14.2 s. **e** FRAP confirmed that nuclear speckled structures did not form and the selected fluorescent region was photobleached in the overexpressed EGFP-YTHDC1-Δ274-294 group. The fluorescence in the bleached area did not recover even after 140 s. **f** Rescue experiments confirmed that lnc668 expression decreased in the si-YTHDC1 group. YTHDC1 overexpression promoted lnc668 expression and reversed the inhibitory effect of si-YTHDC1, whereas YTHDC1-Δ274-294 overexpression did not promote lnc668 expression and did not reverse the inhibitory effect of si-YTHDC1. **g** RNA pull-down experiments showed that the YTHDC1-Δ274-294 mutant failed to bind both lnc668 WT probe and lnc668 m^6^A probe. **h** Nuclear-cytoplasmic separation experiments confirmed that after si-YTHDC1 expression, cytoplasmic lnc668 expression decreased and nuclear lnc668 expression increased. Wild-type YTHDC1 overexpression increased cytoplasmic lnc668 expression and decreased nuclear lnc668 expression, reversing the inhibitory effect of si-YTHDC1. However, YTHDC1-Δ274-294 overexpression did not increase lnc668 expression in the cytoplasm or decrease lnc668 expression in the nucleus, failing to reverse the inhibitory effect of si-YTHDC1. **i** RNA-FISH confirmed that si-YTHDC1 reduced lnc668 expression, with a more significant decrease in the cytoplasm than in the nucleus. Exogenous YTHDC1-WT overexpression after the knockdown of endogenous YTHDC1 led to increased lnc668 expression in the cytoplasm and decreased lnc668 expression in the nucleus, reversing the inhibitory effects of si-YTHDC1. However, YTHDC1-Δ274-294 overexpression did not enhance lnc668 expression in the cytoplasm and was unable to counteract the inhibitory effect of si-YTHDC1.

PONDR and PSPHunter databases displayed that YTHDC1 has LLPS ability. Therefore, the effect of phase-separating YTHDC1 on lnc668 nuclear export was further explored. Immunofluorescence analysis revealed that YTHDC1 formed distinct phase-separated nuclear condensates under TGF-β1 action. The phase separation inhibitor 1,6-hexanediol, an organic compound used in biochemical research to disrupt LLPS, enables the study of the behavior of biomolecules in different phases in cells. Treatment with 1,6-hexanediol led to the disassembly of nuclear condensates (Fig. 4a). An enhanced green fluorescent protein (EGFP)–tagged YTHDC1 (EGFP-YTHDC1) vector was constructed and transfected into MRC-5 cells. Live-cell imaging illustrated that under the same conditions, cells in the EGFP group exhibited no nuclear punctate structures, whereas those in the EGFP-YTHDC1 group displayed nuclear punctate structures. The addition of 1,6-hexanediol significantly reduced the nuclear punctate structures (Fig. 4b). Subsequent live-cell imaging clarified that these nuclear punctate structures fused to form large nuclear punctate structures within 100 s (Fig. 4c), indicating the dynamic liquid nature of the nuclear punctate structures of YTHDC1. The above findings proved that YTHDC1 can form phase separation structures.

Intrinsically disordered regions (IDRs) are needed for protein phase separation formation [33]. PONDR and PSPHunter databases revealed that the 274–294 amino acid fragment is the overlapping IDR in YTHDC1 (Supplementary Fig. 3a, b). An EGFP-YTHDC1-∆274-294 vector with a truncated 274–294 amino acid sequence was constructed and transfected into MRC-5 cells to confirm that the phase separation of YTHDC1 depends on its 274–294 amino acid fragment. The images of fluorescence recovery after photobleaching (FRAP) experiments demonstrated that after photobleaching a region of each granule, fluorescence in the EGFP-YTHDC1 group rapidly recovered within a short period. Fluorescence intensity exceeded 50% of the initial intensity at 14.2 s (Fig. 4d). However, the fluorescence in the EGFP-YTHDC1-Δ274-294 group did not recover to 50% of the initial intensity within a short period, and even at 140 s, fluorescence intensity only recovered to 10% of the initial intensity (Fig. 4e). These results indicated that the 274–294 amino acid fragment participates in YTHDC1 phase separation. The ability of YTHDC1 to form phase separation structures was also proven in 293 T cells (Supplementary Fig. 3c–f). The rescue experiment demonstrated that lnc668 expression in the TGF-β1 group was elevated compared with that in the normal group, and si-YTHDC1 reduced lnc668 expression compared with TGF-β1 treatment. YTHDC1 overexpression elevated lnc668 expression and reversed the inhibitory effect of si-YTHDC1. However, YTHDC1-Δ 274-294 overexpression did not elevate lnc668 expression and also did not reverse the inhibitory effect of si-YTHDC1 (Fig. 4f). The 3’ biotin ssRNA probes without m^6^A modification (lnc668 WT probe) and with m^6^A modification at site 555 (lnc668 m^6^A probe) were chemically synthesized to conduct RNA pull-down assays. The binding of these probes to YTHDC1 and Myc and EGFP-tagged YTHDC1-Δ274-294 mutant protein was then tested. The results showed that lnc668 WT probe bound to YTHDC1, and lnc668 m^6^A probe exhibited stronger binding to YTHDC1. However, the YTHDC1-Δ274-294 mutant, which lacks the key phase separation region (274-294), did not bind to either lnc668 WT probe or lnc668 m^6^A probe (Fig. 4g). These data confirm that YTHDC1 enhances the m^6^A modification of lnc668 through its phase separation ability.

Next, we explored whether YTHDC1 promotes the nuclear export of m^6^A-modified lnc668 through phase separation. The results of the rescue experiment on the nuclear export of lnc668 confirmed that TGF-β1 stimulation increased the expression of lnc668 in the cytoplasm and decreased that in the nucleus. After silencing YTHDC1 expression, cytoplasmic lnc668 expression decreased, and nuclear lnc668 expression increased. The overexpression of wild-type YTHDC1 increased lnc668 expression in the cytoplasm and decreased that in the nucleus, reversing the inhibitory effect of si-YTHDC1. However, the overexpression of YTHDC1-Δ274-294 did not increase lnc668 expression in the cytoplasm or decrease that in the nucleus, failing to reverse the inhibitory effect of si-YTHDC1 (Fig. 4h). The results of the RNA-FISH experiments were consistent with those of the nuclear export rescue assay on lnc668. Compared with those in the control group, the expression levels of lnc668 increased in the nucleus and cytoplasm in the TGF-β1-treated group, with a more significant increase in the cytoplasm than in the nucleus. However, interference with YTHDC1 led to a reduction in lnc668 expression in both compartments, with a more pronounced decrease in the cytoplasm than in the nucleus. The overexpression of exogenous YTHDC1-WT following endogenous YTHDC1 knockdown increased lnc668 expression in the cytoplasm and reduced that in the nucleus, reversing the inhibitory effect of si-YTHDC1. By contrast, the overexpression of YTHDC1-Δ274-294 did not increase lnc668 expression in the cytoplasm and failed to reverse the inhibitory effect of si-YTHDC1 (Fig. 4i). These results indicated that the nuclear export of lnc668 depends on YTHDC1 phase separation.

### Phase-separating YTHDC1 promoted the nuclear export of lnc668 through the SRSF3-ALYREF-XPO5 transport complex

**Fig. 5:**
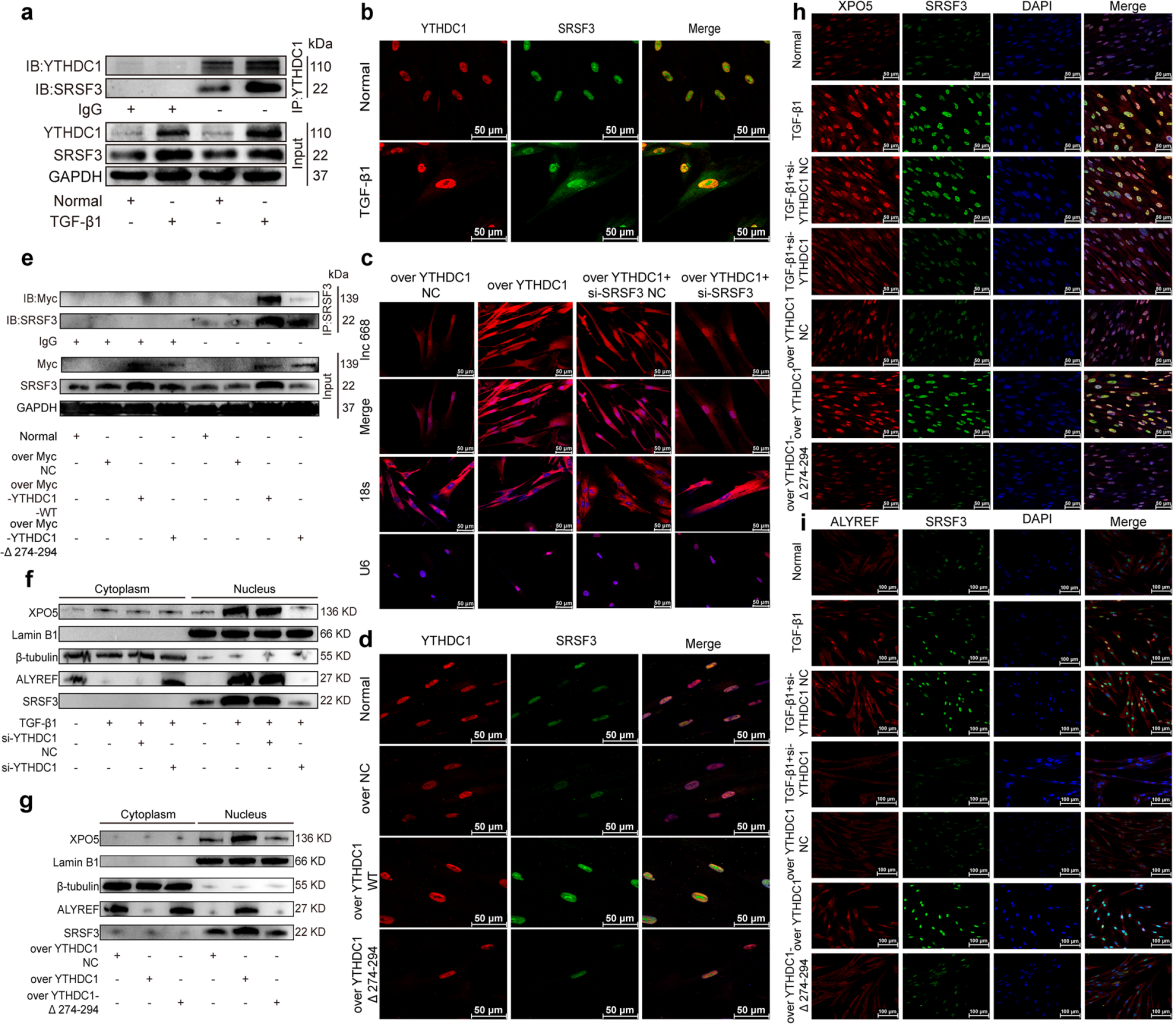
Phase-separating YTHDC1 promoted the nuclear export of lnc668 by forming a transport complex with SRSF3–ALYREF–XPO5. **a** YTHDC1 was enriched in Co-IP experiments. Western blot analysis of the whole cell lysate in the Input group revealed low expression levels of YTHDC1 and SRSF3 in the normal group and high expression levels of YTHDC1 and SRSF3 after TGF-β1 stimulation. Western blot analysis of the lysate enriched with YTHDC1 showed low expression levels of YTHDC1 and SRSF3 in the normal group in the IP group and high expression levels of YTHDC1 and SRSF3 after TGF-β1 stimulation. IP: Immunoprecipitation group; IgG: Negative control group; Input: Positive control group. **b** Immunofluorescence detection revealed that YTHDC1 and SRSF3 colocalized in the nucleus and their colocalization in the TGF-β1 stimulation group increased. **c** RNA-FISH experiments showed that after the overexpression of YTHDC1, the expression of lnc668 increased in the nucleus and cytoplasm, with a more pronounced increase in the cytoplasm than in the nucleus. si-SRSF3 led to a decrease in the expression of lnc668 in the cytoplasm, reversing the promoting effect of YTHDC1 overexpression. **d** Immunofluorescence experiments showed that overexpression of YTHDC1-WT increased YTHDC1 and SRSF3 levels, while YTHDC1-Δ274-294 overexpression decreased both proteins. **e** Co-IP experiments found that the binding of SRSF3 increased after the overexpression of YTHDC1 but decreased after the overexpression of YTHDC1-Δ274-294. **f** The nuclear-cytoplasmic separation experiments confirmed that TGF-β1 stimulation, the nuclear expression of XPO5 and SRSF3 increased, while interference with YTHDC1 led to a decrease in their nuclear expression. Overexpression of YTHDC1 promoted the nuclear import of XPO5 and SRSF3, whereas overexpression of YTHDC1-Δ274-294 lost the ability to promote the nuclear import of XPO5 and SRSF3. **g** The nuclear-cytoplasmic separation experiments confirmed that TGF-β1 stimulation increased the nuclear import of ALYREF and its colocalization with SRSF3, while interference with YTHDC1 reduced this colocalization, causing ALYREF to accumulate in the cytoplasm. Overexpression of YTHDC1 enhanced the nuclear colocalization of ALYREF and SRSF3, whereas overexpression of YTHDC1-Δ274-294 resulted in the loss of the ability to promote the nuclear import of ALYREF, leading to increased localization of ALYREF in the cytoplasm. **h** Immunofluorescence demonstrated that SRSF3 and XPO5 were expressed in the nuclei. After TGF-β1 stimulation, the nuclear expression levels of SRSF3 and XPO5 increased. Interfering with YTHDC1 reduced the nuclear expression of SRSF3 and XPO5, whereas overexpressing YTHDC1 increased the nuclear expression of SRSF3 and XPO5. **i** Immunofluorescence demonstrated that ALYREF was mainly located in the cytoplasm of normal cells, whereas SRSF3 localized in the nucleus. After stimulation with TGF-β1, the nuclear expression of ALYREF increased and the cytoplasmic expression of ALYREF decreased. The nuclear expression of SRSF3 also increased. Interference with YTHDC1 led to a reduction in the colocalization of ALYREF and SRSF3 in the nucleus. By contrast, the overexpressed YTHDC1 enhanced the nuclear colocalization of these two proteins, whereas the overexpressed YTHDC1-Δ274-294 resulted in the decreased nuclear colocalization of ALYREF and SRSF3 proteins.

The interaction of YTHDC1 and serine/arginine-rich splicing factor 3 (SRSF3) has been reported to mediate the nuclear export of m^6^A-modified mRNA [34, 35]. However, whether their interaction promotes lncRNA nuclear export remains unclear. Therefore, we further explored this point. In the Co-IP experiment, the YTHDC1 protein was enriched. The Western blot analysis of the whole cell lysate from the input group revealed low expression levels of YTHDC1 and SRSF3 in the normal group. By contrast, high expression levels of YTHDC1 and SRSF3 were observed after TGF-β1 stimulation. The Western blot analysis of the lysate enriched with YTHDC1 protein showed a direct interaction between YTHDC1 and SRSF3 in MRC-5 cells in the IP group and found that TGF-β1 enhanced this direct interaction (Fig. 5a). Moreover, immunofluorescence analysis revealed that YTHDC1 and SRSF3 colocalized in the nucleus and their colocalization increased in the TGF-β1 stimulation group compared with in the control group (Fig. 5b). This finding suggested that YTHDC1 may facilitate the nuclear export of lnc668 via SRSF3. RNA-FISH experiments showed that after the overexpression of YTHDC1, the expression of lnc668 increased in the nucleus and cytoplasm, with a more pronounced increase in the cytoplasm than in the nucleus. Interference with SRSF3 led to a decrease in its expression in the cytoplasm, reversing the promoting effect of YTHDC1 overexpression (Fig. 5c). This result indicated that the nuclear export of lnc668 is dependent on SRSF3. Immunofluorescence analysis revealed that, compared to the control group, overexpression of YTHDC1-WT led to an increase in the expression of both YTHDC1 and SRSF3, while overexpression of YTHDC1-Δ274-294 resulted in a decrease in the expression levels of both proteins (Fig. 5d). The Co-IP experiment found that SRSF3 bound strongly with exogenously expressed YTHDC1-WT, while the interaction with YTHDC1-Δ274-294 was weak (Fig. 5e). This finding indicated that the promotion of lnc668 nuclear export by SRSF3 also depends on the phase separation of YTHDC1. SRSF3 can interact with nuclear pore complexes (NPCs) to promote the transport of mature mRNA from the nucleus to the cytoplasm [36]. Nuclear export receptors can facilitate the bidirectional transport of molecules between the nucleus and cytoplasm through NPCs. Nuclear adaptors, as carriers in nucleocytoplasmic transport, bind to nuclear export receptors and cargo molecules (such as mRNA and lncRNA) to form a transport complex. This complex is transported through NPCs. Therefore, the classic nuclear export receptor Aly/REF export factor (ALYREF) and the nuclear adaptor exportin-5 (XPO5) were selected to study the nuclear export of lnc668. The immunofluorescence experiment preliminarily demonstrated that ALYREF and XPO5 were highly expressed in the nuclei of the TGF-β1 group and their functions depended on the phase separation of YTHDC1 (Supplementary Fig. 3g, h). Nuclear-cytoplasmic separation experiments were performed to validate the localization of ALYREF, XPO5, and SRSF3. XPO5 and SRSF3 were localized in the nucleus of MRC-5 cells, while ALYREF was localized in the cytoplasm. After TGF-β1 stimulation, the nuclear expression of XPO5 and SRSF3 increased, while interference with YTHDC1 led to a decrease in their nuclear expression. Overexpression of YTHDC1 promoted the nuclear import of XPO5 and SRSF3, whereas overexpression of YTHDC1-Δ274-294 lost the ability to promote the nuclear import of XPO5 and SRSF3. ALYREF was primarily localized in the cytoplasm of MRC-5 cells, but after TGF-β1 stimulation, its nuclear expression increased, along with an increase in its nuclear co-localization with SRSF3. Interference with YTHDC1 reduced the nuclear co-localization of ALYREF and SRSF3, causing ALYREF to accumulate in the cytoplasm. On the other hand, overexpression of YTHDC1 enhanced the nuclear co-localization of these two proteins, with a corresponding decrease of ALYREF in the cytoplasm. Overexpression of YTHDC1-Δ274-294 resulted in the loss of the ability to promote the nuclear import of ALYREF, with an increase in its cytoplasmic localization (Fig. 5f, g). Next, we further explored the expression levels and localization relationship with SRSF3 of ALYREF and XPO5. Immunofluorescence detection revealed that XPO5 and SRSF3 were expressed in the nuclei of MRC-5 cells. After stimulation with TGF-β1, their nuclear colocation increased. Interference with YTHDC1 led to a decrease in the colocation of XPO5 and SRSF3 in the nucleus, whereas the overexpression of YTHDC1 promoted their nuclear colocation. By contrast, the overexpression of YTHDC1-Δ274-294 reduced the nuclear colocation of XPO5 and SRSF3. A further immunofluorescence experiment revealed that ALYREF was mainly located in the cytoplasm, whereas SRSF3 localized in the nucleus. After stimulation with TGF-β1, the nuclear expression of ALYREF increased and the cytoplasmic expression of ALYREF decreased, whereas the nuclear expression of SRSF3 increased. Interference with YTHDC1 led to a reduction in the colocalization of ALYREF and SRSF3 in the nucleus. By contrast, the overexpression of YTHDC1 enhanced the nuclear colocalization of these two proteins, whereas the overexpression of YTHDC1-Δ274-294 resulted in the decreased nuclear colocalization of the ALYREF and SRSF3 proteins (Fig. 5h, i). The above findings suggested that phase-separating YTHDC1 may form a complex with SRSF3, ALYREF, and XPO5 to promote the nuclear export of m^6^A-modified lnc668 during fibroblast-to-myofibroblast differentiation.

### lnc668 enhanced PICALM mRNA stability to promote pulmonary fibrogenesis depending on YTHDC1 phase separation

**Fig. 6:**
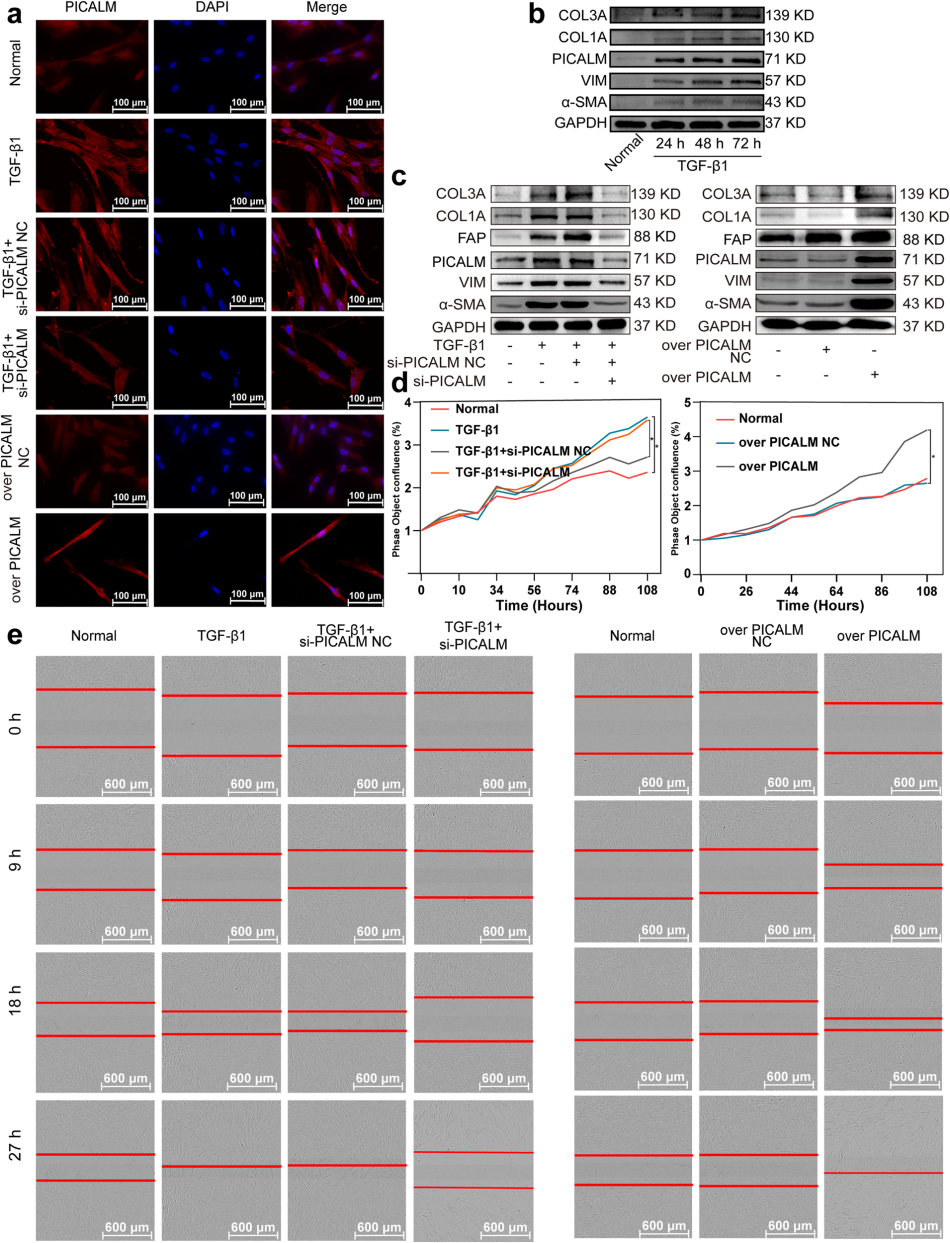
PICALM functioned as a profibrotic factor to promote pulmonary fibrogenesis. **a** Immunofluorescence images revealed that PICALM expression increased in the nucleus and cytoplasm under the TGF-β1 or PICALM overexpression treatment and decreased under the si-PICALM treatment. **b** COL1A, COL3A, VIM, α-SMA, and PICALM expression levels increased with the prolongation of the time of TGF-β1 action. **c** Western blot unveiled that PICALM, COL1A, COL3A, FAP, VIM, and α-SMA expression levels were decreased by si-PICALM and increased by PICALM overexpression. **d** IncuCyte S3 live-cell analysis confirmed that myofibroblast proliferation was inhibited by si-PICALM and promoted by PICALM overexpression. **e** Scratch assays confirmed that si-PICALM reduced cell migration, whereas PICALM overexpression enhanced cell migration.

Given that approximately 40% of lncRNAs exert their regulatory function by specifically interacting with their host genes, the effect of lnc668 on its host gene PICALM was further examined. Immunofluorescence images demonstrated that PICALM was present in the nucleus and cytoplasm in normal MRC-5 cells. Comparison with the normal group showed that after TGF-β1 stimulation, PICALM expression increased in the nucleus and cytoplasm. Compared with the TGF-β1 treatment, interference with PICALM reduced PICALM expression in the nucleus and cytoplasm, whereas the overexpression of PICALM increased PICALM expression in the nucleus and cytoplasm (Fig. 6a). Western blot analysis illustrated that with the prolonged stimulation of MRC-5 cells by TGF-β1, the expression of PICALM, as well as VIM, α-SMA, COL1A, and COL3A, gradually increased (Fig. 6b). The expression levels of these proteins were decreased by si-PICALM and increased by PICALM overexpression (Fig. 6c). IncuCyte S3 live-cell analysis demonstrated that the proliferative capacity of myofibroblasts was inhibited by si-PICALM and promoted by PICALM overexpression (Fig. 6d). Scratch assays proved that si-PICALM reduced cell migration ability, whereas PICALM overexpression enhanced cell migration (Fig. 6e). The above results indicated that PICALM can promote fibroblast-to-myofibroblast differentiation, leading to fibrogenesis.

**Fig. 7:**
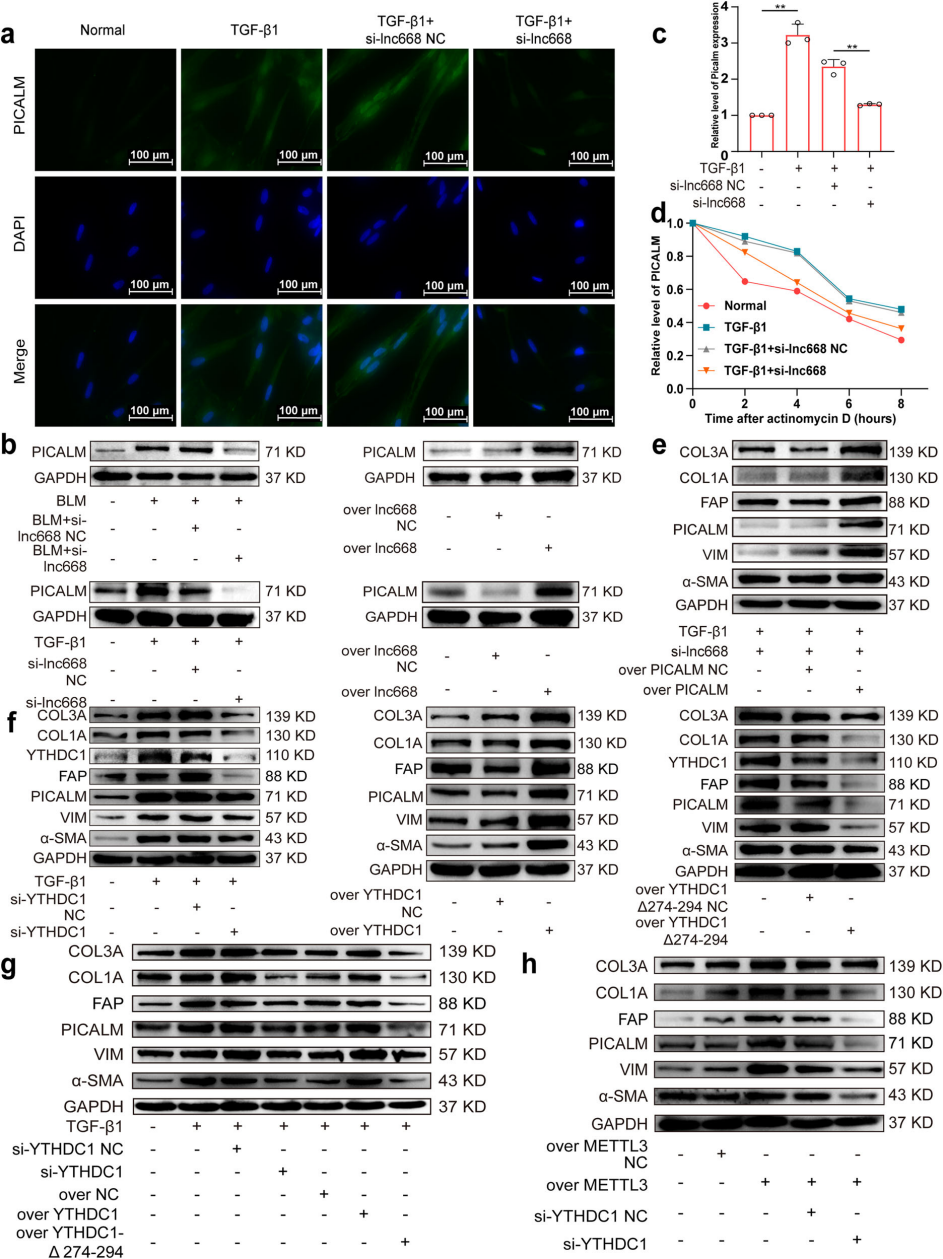
lnc668-PICALM-mediated pulmonary fibrogenesis depended on YTHDC1 phase separation. **a** Immunofluorescence detection uncovered that si-lnc668 reduced the expression of PICALM in cells. **b** PICALM expression was inhibited by si-lnc668 and promoted by overexpression in vivo and in vitro. **c** qRT-PCR showed that si-lnc668 reduced PICALM mRNA expression. **d** Half-life analysis revealed that PICALM mRNA stability was enhanced by TGF-β1 but reduced by si-lnc668. **e** Rescue experiments confirmed that si-lnc668 reduced the expression levels of PICALM, FAP, VIM, α-SMA, COL1A, and COL3A. PICALM overexpression increased the expression levels of these proteins. **f** Western blot analysis confirmed that si-YTHDC1 significantly reduced the elevated levels of YTHDC1, PICALM, FAP, VIM, α-SMA, COL1A, and COL3A induced by TGF-β1 stimulation. The overexpression of YTHDC1 increased the expression levels of PICALM, FAP, VIM, α-SMA, COL1A, and COL3A. However, YTHDC1-Δ274-294 overexpression did not increase the expression levels of YTHDC1, PICALM, FAP, VIM, α-SMA, COL1A, and COL3A. **g** Western blot rescue experiments indicated that si-YTHDC1 led to the decreased expression levels of FAP, VIM, α-SMA, PICALM, COL1A, and COL3A. Conversely, overexpressed YTHDC1 elevated the expression levels of these proteins, counteracting the suppressive effect of si-YTHDC1. However, the YTHDC1-∆274-294 overexpression mutant failed to increase the expression levels of these proteins and did not counteract the suppressive effect of si-YTHDC1. **h** Rescue experiments confirmed that METTL3 overexpression increased the expression levels of FAP, VIM, α-SMA, COL1A, and COL3A. si-YTHDC1 reduced the expression of lnc668, reversing the effect of METTL3 on the above proteins.

Immunofluorescence images confirmed that compared with TGF-β1 treatment, interference with lnc668 reduced PICALM expression in the nucleus and cytoplasm (Fig. 7a). Western blot analysis indicated that interference with lnc668 decreased PICALM expression levels, whereas lnc668 overexpression increased PICALM expression levels in vivo and in vitro (Fig. 7b). qRT-PCR results demonstrated that lnc668 knockdown significantly reduced PICALM mRNA expression (Fig. 7c). Half-life analysis showed that the stability of PICALM at the mRNA level was enhanced by TGF-β1 stimulation but reduced by si-lnc668 (Fig. 7d). Rescue experiments illustrated that si-lnc668 treatment weakened the expression levels of PICALM, FAP, VIM, α-SMA, COL1A, and COL3A. PICALM overexpression promoted the expression levels of these proteins and reversed the inhibitory effect of si-lnc668 (Fig. 7e). The above results indicated that lnc668 executes its anti-pulmonary fibrosis function by specifically interacting with PICALM.

Western blot analysis unveiled that the action of si-YTHDC1 decreased the expression levels of FAP, VIM, α-SMA, PICALM, YTHDC1, COL1A, and COL3A relative to that of TGF-β1. The expression levels of these proteins increased under the action of YTHDC1 overexpression (Fig. 7f) but were not enhanced by YTHDC1-∆274-294 overexpression (Fig. 7f). Furthermore, comparison with the normal group showed that the expression level of lnc668 decreased rather than increased after YTHDC1-∆274-294 overexpression (Supplementary Fig. 2h). Rescue experiments using Western blot analysis revealed that the expression levels of FAP, VIM, α-SMA, PICALM, COL1A, and COL3A decreased under si-YTHDC1 treatment. However, the overexpression of YTHDC1 increased the expression of these proteins, reversing the inhibitory effect of si-YTHDC1. By contrast, the overexpression of the YTHDC1-∆274-294 mutant did not increase the expression levels of these proteins or reverse the inhibitory effect of si-YTHDC1 (Fig. 7g). Western blot rescue experiments confirmed that the expression levels of FAP, VIM, α-SMA, COL1A, and COL3A elevated under METTL3 overexpression. After silencing YTHDC1 expression, the levels of these proteins decreased, reversing the trend under METTL3 overexpression (Fig. 7h). These findings indicated that lnc668–PICALM-mediated pulmonary fibrogenesis depends on YTHDC1 phase separation.

### Evaluation of the potential of lnc668 as a therapeutic target in mice and patients

**Fig. 8:**
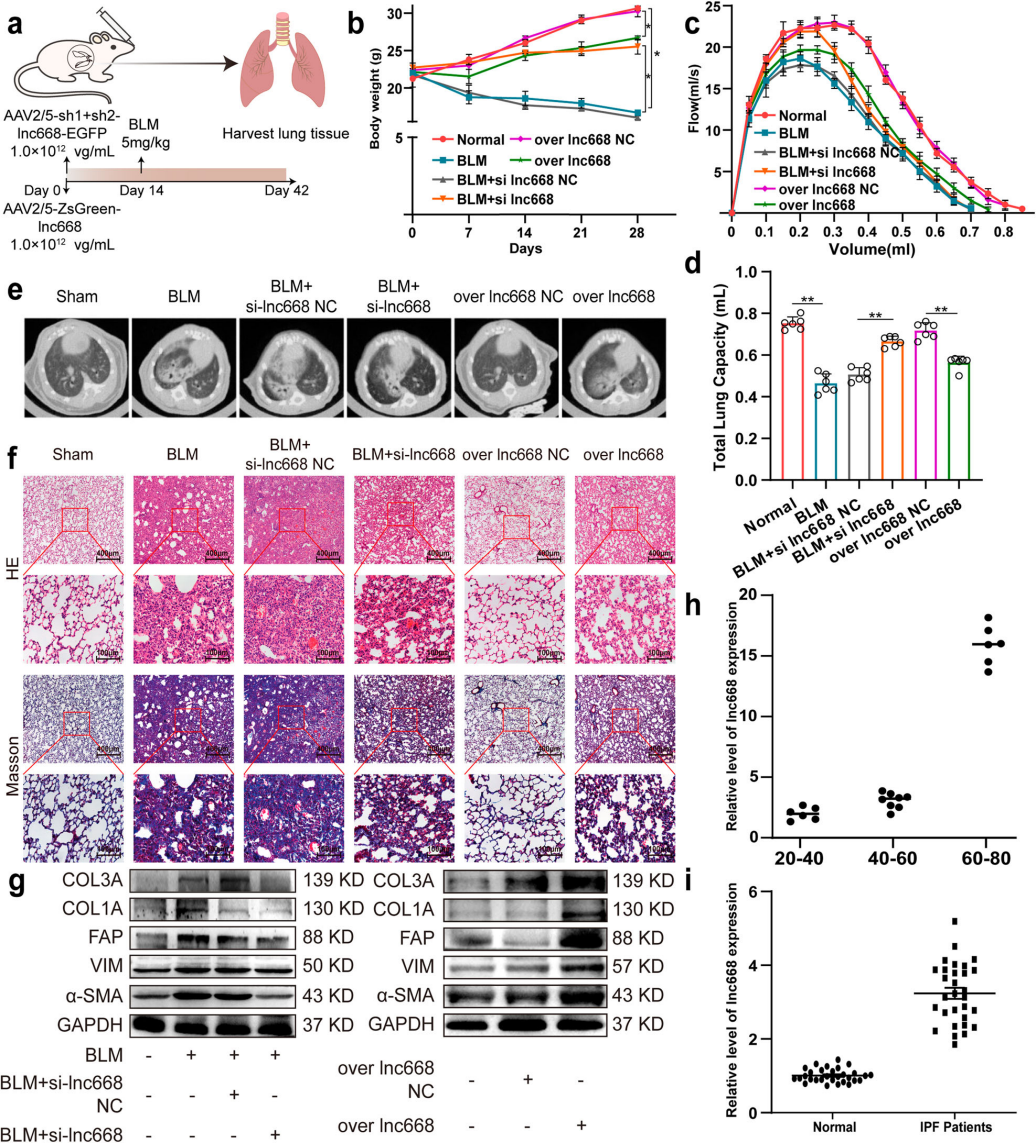
Evaluation of lnc668 as a therapeutic target for pulmonary fibrosis. **a** lnc668 interference fragment and overexpression adenoviral vector were administered to mice via intratracheal injection. **b** Body weight loss was obvious in the BLM and lnc668 overexpression groups. The si-lnc668 group did not experience remarkable weight loss. **c** Lung function in the si-lnc668 group improved compared with that in the BLM group. Similar to BLM treatment, lnc668 overexpression decreased lung function. **d** Total lung capacity after si-lnc668 treatment increased compared with that after BLM treatment. Similar to BLM treatment, lnc668 overexpression decreased total lung capacity. **e** MicroCT imaging system for small animals revealed that interfering with lnc668 alleviated the pulmonary fibrosis caused by BLM. lnc668 overexpression accelerated pulmonary fibrosis. **f** HE and Masson staining demonstrated that alveolar walls thickened, alveolar structures became disordered, and collagen deposition increased in the BLM model group. si-lnc668 attenuated the above symptoms. lnc668 overexpression caused symptoms similar to those caused by BLM treatment. **g** Western blot confirmed that compared with BLM treatment, interference with lnc668 reduced the expression levels of FAP, α-SMA, COL1A, COL3A, and VIM. lnc668 overexpression increased the expression levels of these proteins. **h** lnc668 expression in the blood of patients with IPF was significantly higher than that in healthy individuals. **i** qRT-PCR proved that lnc668 expression in the blood of normal people increased with the increase in age.

Finally, we evaluated lnc668 as a potential therapeutic target in the BLM-treated mouse model of pulmonary fibrosis and patients with IPF. The lnc668 interference fragment and lnc668 overexpression plasmid were packaged into an adenoviral vector, each with a titer of 1.0 × 10¹² vg/mL, and administered to the lungs of C57BL/6 mice via tracheal nebulization (Fig. 8a). Mouse body weight decreased significantly in the BLM and lnc668 overexpression groups but increased in the si-lnc668 group (Fig. 8b). Lung function declined in the BLM and lnc668 overexpression groups but improved after lnc668 interference (Fig. 8c). Total lung capacity decreased in the BLM and lnc668 overexpression groups but recovered after lnc668 interference (Fig. 8d). The microCT imaging system for small animals depicted that in the BLM and lnc668 overexpression groups, lung texture coarsened with grid-like shadows, accompanied with alterations in lung parenchymal structure and decreased lung tissue elasticity. Interference with lnc668 somewhat alleviated lung abnormalities (Fig. 8e). The staining of pathological slices revealed prominent lung fibrosis in the BLM and lnc668 overexpression groups, characterized by extracellular matrix deposition and alveolar structure destruction, which was alleviated in the si-lnc668 group (Fig. 8f). Western blot results demonstrated that BLM and lnc668 overexpression significantly elevated the levels of fibrosis- and differentiation-related proteins. Interference with lnc668 decreased the expression levels of these proteins (Fig. 8g). The in vivo and in vitro findings indicated that interference with lnc668 can attenuate pulmonary fibrogenesis via inhibiting fibroblast-to-myofibroblast differentiation.

Next, the clinical significance of lnc668 in patients with IPF was assessed. qRT-PCR showed that the expression of lnc668 in the blood of patients with IPF was significantly higher than that in healthy individuals (Fig. 8h). Interestingly, the expression of lnc668 increased with age in a healthy population (Fig. 8i). Given that pulmonary fibrosis is an age-related disease, lnc668 may be a diagnostic biomarker for age-induced pulmonary fibrosis. All the above findings suggested that lnc668 can be a therapeutic target for pulmonary fibrosis.

## Discussion

**Fig. 9:**
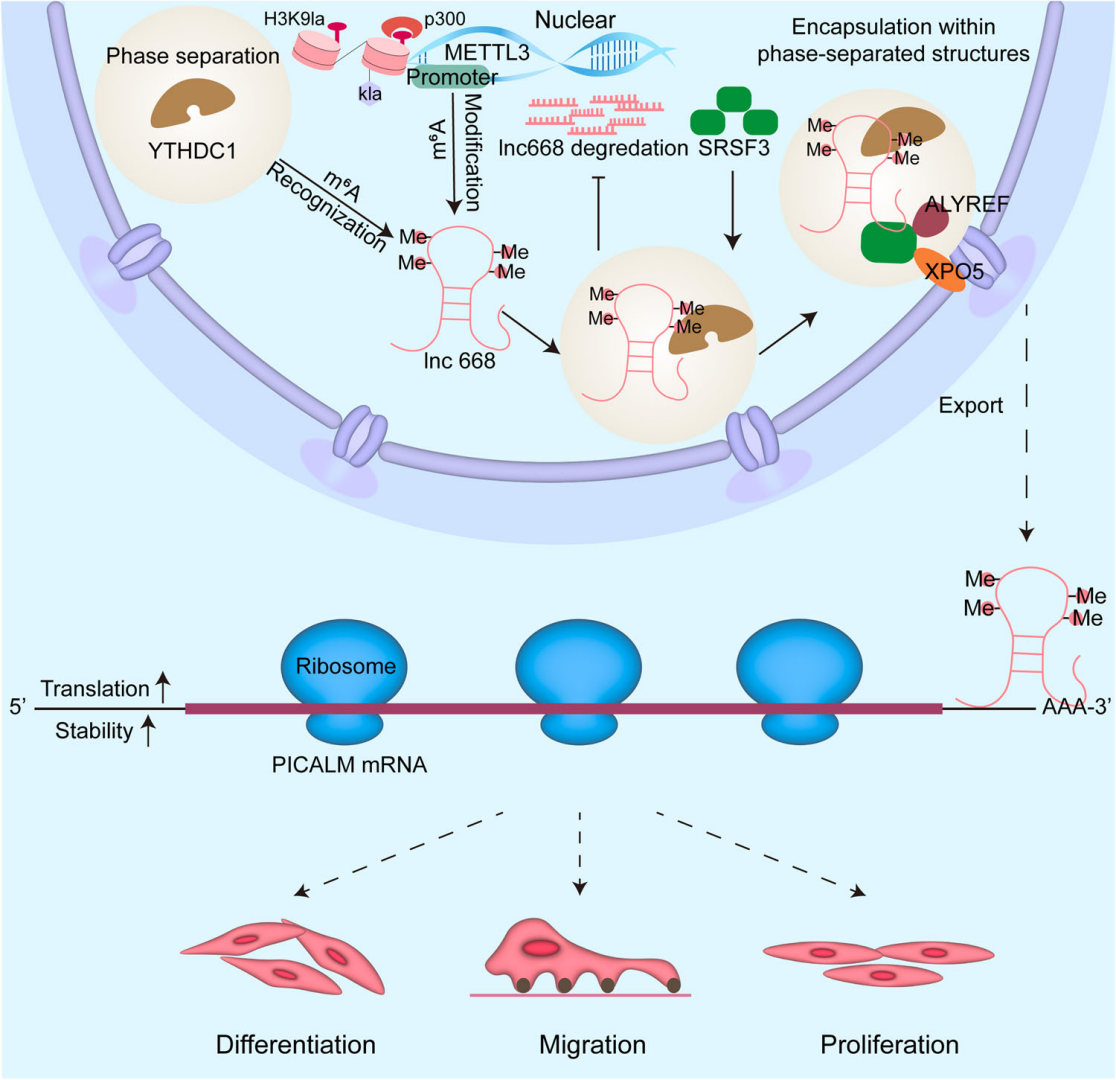
The mechanism of lnc668 acts as a pro-fibrotic factor to promote pulmonary fibrosis.

Our study elucidated that the nuclear export of lnc668 exacerbated pulmonary fibrosis by activating fibroblast-to-myofibroblast differentiation. Mechanistic research showed that histone H3K9 lactylation enriched in the promoter region of METTL3 to enhance METTL3 transcription, leading to the m^6^A modification of lnc668. Meanwhile, YTHDC1 recognized the m^6^A modification of lnc668 to elevate the METTL3-mediated m^6^A modification of lnc668. Subsequently, phase-separating YTHDC1 promoted the nuclear export of m^6^A-modified lnc668. In this process, it formed a NPC with SRSF3, ALYREF, and XPO5 to assist the translocation of m^6^A-modified lnc668 from the nucleus to the cytoplasm. After nuclear export, lnc668 facilitated the translation and stability of its host gene PICALM to activate fibroblast-to-myofibroblast differentiation, leading to the aggravation of pulmonary fibrosis, which also depended on the phase separation of YTHDC1 (Fig. 9).

Generated lncRNAs can localize in the nucleus or cytoplasm to exert their specific functions. For example, the lncRNA NEAT1 is mainly enriched in the nucleus but also found in the cytoplasm. In the nucleus, the induction of the lncRNA NEAT1 can relocate SFPQ from the IL8 promoter to paraspeckles, leading to the transcriptional activation of IL8 upon immune stimuli [36]. In the cytoplasm, Pinin mediates the glucose-stimulated nuclear export of the lncRNA NEAT1 to promote breast cancer growth and metastasis by regulating glycolysis [37]. Cytoplasmic lncRNAs, which require nuclear output, usually contain specific nuclear export signals that are recognized and bound by nuclear export proteins [38, 39]. These proteins can facilitate their transport from the nucleus to the cytoplasm [40–42]. Khan et al. identified a cytoplasmic accumulation region (CAR-N) in the intronless lncRNA NKILA. CAR-N removal leads to the strong nuclear retention of the lncRNA NKILA, whereas CAR-N insertion promotes the export of cDNA transcripts. The lncRNA NKILA lacking CAR-N cannot inhibit breast cancer cell migration [42]. The mitochondrial lncRNA RMRP is encoded by nuclear DNA, and the RNA-binding proteins HuR and GRSF1 modulate its nuclear export and mitochondrial localization. The loss of GRSF1 decreases the mitochondrial levels of RMRP, in turn suppressing oxygen consumption rates and modestly reducing mitochondrial DNA replication priming [43]. Numerous cytoplasmic lncRNAs are involved in pulmonary fibrosis. The cytoplasmic lncRNA DACH1 binds to the splicing factor SRSF1, which suppresses the accumulation of CTNNB1 (β-catenin), thereby inhibiting lung fibroblast activation and extracellular matrix deposition [44]. The macrophage-derived lncRNA MSTRG.91634.7 upregulates PINK1, a key protein in mitochondrial selective autophagy. This upregulation inhibits the formation of silica-induced lung inflammation and fibrosis [45]. However, how these lncRNAs translocate from the nucleus to the cytoplasm to exert their profibrotic functions remains unexplored. Our present study found that that the nuclear export of m^6^A-modified lnc668 through the NPCs SRSF3, ALYREF, and XPO5 depended on YTHDC1 phase separation.

Numerous studies have shown that m^6^A modification is involved in the advancement of diverse diseases, such as diabetes and cancer, via regulating DNA replication, alternative splicing, and mRNA export. In the keratinocytes of diabetic skin, a decrease in the m^6^A reader YTHDC1 inhibits autophagy via the acceleration of the nuclear mRNA decay of SQSTM1, resulting in the impaired migration abilities of keratinocytes and delayed healing of wounds [46]. YTHDC1 can form a complex with SLC12A5 to upregulate HOXB13, leading to the promotion of prostate cancer progression [47, 48]. The lncRNA ZNRD1-AS1 promotes malignant lung cell proliferation, migration, and angiogenesis via the miR-942–TNS1 axis and is positively regulated by the m^6^A reader YTHDC2 [49, 50]. However, m^6^A-modified lncRNAs in pulmonary fibrosis remain unstudied. Our study found that TGF-β1 enhanced the enrichment of H3K9la at the METTL3 promoter region to promote the transcription of METTL3. The inhibition of histone acetyltransferase p300 decreased the level of H3K9la, leading to the downregulation of METTL3 mRNA levels. This effect suggests that histone lactylation assisted the m^6^A modification of lnc668 in pulmonary fibrosis. Subsequently, we further elucidated that m^6^A modification assisted lnc668 nuclear export. We speculate that m^6^A modification affected the secondary structure of lnc668, exposing binding sites for nuclear export proteins, such as YTHDC1, SRSF3, ALYREF, and XPO5, to form NPCs, and YTHDC1 phase separation stabilized NPCs, which facilitated the trafficking of m^6^A-modified lnc668 to the cytoplasm.

NPCs function as a gatekeeper, controlling transport between the nucleus and cytoplasm. The central channel of NPCs contains hundreds of proteins called FG-nucleoporins with IDRs, which are the essential regions for phase separation [51]. Although the structure of the NPC scaffold has been resolved in remarkable detail, the actual transport machinery remains poorly understood [50]. YTHDC1 exhibits phase separation capability, and m^6^A is required for YTHDC1 to undergo phase separation and form nuclear YTHDC1-m^6^A condensates (nYACs). The number of nYACs in acute myeloid leukemia cells is higher than that in normal hematopoietic stem and progenitor cells [52]. YTHDC1 is also known to participate in mRNA nuclear export [53]. It promotes m^6^A-modified SMAD3 nuclear export to augment the TGF-β signaling cascade leading to triple negative breast cancer metastasis [54]. However, whether lncRNA nuclear export depends on YTHDC1 phase separation and whether phase separation directly affects the occurrence and development of pulmonary fibrosis remain unproven. Our present study clarified that YTHDC1 phase separation not only aids its recognition and binding of the m^6^A-modified lnc668 in the nucleus but also promotes the nuclear export of lnc668. Although our study discovered that METTL3 overexpression increased m^6^A modification, silencing YTHDC1 did not enhance the nuclear export of lnc668. Additionally, the nuclear export of lnc668 could still occur with the addition of exogenous wild-type YTHDC1 after the interference of endogenous YTHDC1. However, nuclear export could not proceed with the addition of YTHDC1-Δ274-294. These findings indicated that although YTHDC1 phase separation was crucial for recruiting m^6^A-modified lnc668 to phase-separated structures, the presence of YTHDC1 alone was insufficient for the nuclear export of lnc668. The nuclear export of lnc668 still needed SRSF3, ALYREF, and XPO5 recruited through YTHDC1 phase separation for the structural integrity of NPCs. Therefore, the nuclear condensate formation of YTHDC1 is a necessary but insufficient condition for lnc668 nuclear export. After nuclear export, cytoplasmic lnc668 promoted the translation and stability of PICALM mRNA, leading to increased levels of differentiation-related and fibrotic proteins, such as FAP, α-SMA, COL1A, COL3A, and VIM, thus contributing to the progression of pulmonary fibrosis.

Our study first clarified three issues: one, H3K9la promotes the m^6^A modification of lnc668 by enhancing the transcription of METTL3. Two, without YTHDC1 phase separation, lnc668 cannot be transported to the cytoplasm, even with increased levels of m^6^A-modified lnc668. Three, the profibrotic function of lnc668 via PICALM depends on YTHDC1 phase separation. Collectively, YTHDC1 phase separation drives the nuclear export of lnc668 through the NPC SRSF3–ALYREF–XPO5. Subsequently, cytoplasmic lnc668 promotes fibroblast-to-myofibroblast differentiation via PICALM to exacerbate pulmonary fibrosis. Targeting this pathway will be an excellent strategy for the treatment of human pulmonary fibrosis.

## Methods

All animal experiments followed the guidelines and protocols approved by the Research Ethics Committee of Affiliated Hospital, Binzhou Medical university. the Research Ethics Committee of Affiliated Hospital approved the use of human samples and research for this study (approval number: KYLL-2022-175). Written informed consent was obtained by all participants.

### IPF patients

Blood samples were collected from 30 patients with IPF at the affiliated hospital of Binzhou Medical University (Binzhou and Yantai, China). The IPF patients were diagnosed in accordance with the American Thoracic Society/European Respiratory Society consensus criteria. The 30 matched control healthy individuals were selected based on the IPF patients age and gender. All participants provided written informed consent, and the study was approved by the Ethics Committee of Binzhou Medical University.

### Animal model and treatment

This experiment was approved by the Animal Ethics Committee of Binzhou Medical College. Eight-week-old C57BL/6 mice were provided by Jinan Pengyue Company and divided into the following groups according to experimental requirements: sham operation group, BLM model group, BLM + si-lnc668/NC group, BLM + si-lnc668 group, overexpressed lnc668/NC group, and overexpressed lnc668 treatment group, with 10 mice in each group. Anesthesia was induced by intraperitoneal injection of 4% chloral hydrate (10 mg/kg). The sham operation group received an equivalent volume of saline as a control. To enhance lnc668 expression, the mice were injected with 50 μL of AAV via intratracheal instillation, Packages of recombinant adeno-associated virus (rAAVs) serotype 5 vectors were provided by HanBio Technology Co., Ltd, (Shanghai, China). Shortly, the lnc668 gene of over-expression AAV (AAV2/5-ZsGreen-lnc, 1.0 ×10^12^ vg/mL) was synthesized, while an AAV with empty vector expressing GFP alone (AAV-empty, 1.0 ×10^12^ vg/mL) was used as the control. However, to decrease lnc668 expression, the si- lnc668 gene of AAV (AAV2/5-sh1+sh2-lnc668-EGFP, 1.0 × 10^12^ vg/mL) was synthesized, while an AAV with si-NC expressing GFP (AAV2/5-shNC-EGFP, 1.0 × 10^12^ vg/mL) was used as the control. On day 0, AAV2/5-sh1+sh2-lnc668-EGFP administered to the lungs of mice in the BLM + si-lnc668 group via tracheal nebulization using a Penn-Century MicroSprayer (PennCentury Inc., Wyndmoor, PA, USA). AAV2/5-ZsGreen-lnc668 administered via tracheal nebulization to the lungs of mice in the overexpressed lnc668 treatment group. Fourteen days later, mice treated with bleomycin received 4.3 mg/kg of bleomycin via tracheal spray. Lung tissues from all groups were collected on day 28 after bleomycin treatment and immediately frozen in liquid nitrogen for further study. This experiment was approved by the Animal Experiment Ethics Committee of Binzhou Medical University.

### Lung function measurement

Mice were anesthetized with 10 mg/kg of 4% chloral hydrate administered via intraperitoneal injection. A plastic cannula was used to connect a metal adaptor to an endotracheal tube inserted into the mouse trachea, and the other end of the plastic cannula was then connected to a pulmonary mechanics apparatus (DSI Buxco, USA). Mechanical ventilation was set with a respiratory rate of 150 breaths/min, a tidal volume of 10 mL/kg, and PEEP was set at 3 cm H2O. Negative pressure forced expiration (NPFE) maneuver was then applied. The mouse lungs were inflated to 30 cm H2O for 2 s, followed by connection of the airway to a negative pressure reservoir (−50 cm H2O) for 2 s. Forced vital capacity (FVC) was calculated based on the flow-volume loop generated during lung deflation.

### MicroCT measurement

The mice were intraperitoneally injected with 4% chloral hydrate at a dosage of 10 mg/kg to induce anesthesia. Subsequently, they were transferred to a scanning bed for in vivo MicroCT imaging (PerkinElmer, USA). X-ray parameters were adjusted to 90 kV and 88 μA. The MicroCT image resolution was set at 36 mm FOV, with an exposure time of 4 minutes. Two-dimensional cross-sectional images were obtained using imaging software (SimpleViewer). Three-dimensional reconstruction was performed by using Analyze 11.0 software (AnalyzeDirect, USA).

### Hematoxylin-eosin (H&E) and Masson staining

Mouse lung tissues fixed with 4% paraformaldehyde were dehydrated, embedded in paraffin overnight, and then sectioned by using a Leica (RM2255) microtome into 4 μm slices. The sections were stained with H&E and Masson’s trichrome staining kit (Solarbio, China). Stained sections were dehydrated with absolute ethanol, made transparent with xylene, mounted with neutral gum, and sealed with cover glass. Lung tissue sections from each group were observed under a light microscope.

### Cell model and treatment

Human fetal lung fibroblasts (MRC-5 cell line) were obtained from the American Type Culture Collection (ATCC). They were cultured in Minimum Essential Medium (MEM, Gibco, 11090-081) supplemented with 10% Fetal Bovine Serum (FBS, Gibco), 1% GlutaMax (Gibco, 35050061), 1% Non-Essential Amino Acids (NEAA, Gibco, 11140050), 1% Sodium pyruvate (Gibco, 11360070), and 1% Penicillin-Streptomycin solution (Sigma-Aldrich), and placed in a cell culture incubator at 37 °C with 5% CO2. Cells were treated with 5 ng/mL TGF-β1 (Gibco, PHG9202) for different durations according to experimental requirements. According to experimental requirements, cells were divided into the following groups: normal group, TGF-β1 treatment group, TGF-β1 + si-lnc668/NC group, TGF-β1 + si-lnc668 group, overexpressed lnc668/(NC) group, and overexpressed lnc668 treatment group. The control group cells were treated with serum-free medium for 72 hours. Cells in the TGF-β1 induced group were treated with 5 ng/mL TGF-β1 for 72 hours. Cells in the si-lnc668 treatment group were first treated with 10 μg/mL lnc668 small interfering RNA (siRNA) fragments (RiboBio, Guangzhou, China) for 6 hours, followed by treatment with 5 ng/mL TGF-β1 for 72 hours. Cells in the lnc668 overexpression group were first treated with 1 ng/mL overexpressed lnc668 (HanBio, Shanghai, China) for 6 hours, followed by treatment with serum-free medium for 72 hours.

### Western blot

MRC-5 cells or lung tissues collected were lysed using radioimmunoprecipitation assay (RIPA) buffer supplemented with phenylmethylsulfonyl fluoride (PMSF) at a ratio of RIPA buffer to PMSF of 100:1. The protein concentration was determined using a bicinchoninic acid protein assay kit (Coolaber, China). The proteins were separated by sodium dodecyl sulfate-polyacrylamide gel electrophoresis (SDS-PAGE) and transferred onto polyvinylidene fluoride (PVDF) membranes. The membranes were blocked with 5% skim milk for 2 hours and then incubated overnight at 4 °C with anti-COL1A, anti-COL3A, anti-VIM, anti-α-SMA, anti-FAP, anti-GAPDH, anti-PICALM, anti-YTHDC1, anti-METTL3, and anti-METTL14 polyclonal antibodies. After washing the membranes three times with 1× Tris buffer Saline Tween, they were incubated with the corresponding secondary antibodies at room temperature for 1 hour. Protein expression was detected using an enhanced chemiluminescence kit (Spark Jade, China). Protein bands were quantitatively analyzed using Image J software. The product numbers and brands of antibodies were listed in Supplementary Table1.

### Real-time cellular proliferation and migration analyses

A proliferation plate containing 1 × 10^5^ cells (Agilent, 5,469,830,001) was placed in the IncuCyte S3 Live-Cell Analysis System (Essen BioScience, USA) for real-time dynamic observation, with automatic recording of cell proliferation over time. A migration plate containing 5 × 10^4^ cells (Corning, 05665817001) was subjected to scratch treatment and placed in the IncuCyte S3 system to automatically record scratch closure. The experiment was scanned using the IncuCyte S3 software and analyzed using IncuCyte 2021 A software. The cell index representing the number of proliferating or migrating cells was calculated using the RTCA system from ACEA Biosciences (ACEA Biosciences, China).

### qRT-PCR

Total RNA was isolated from cells or tissues using the AG RNAex Pro kit (Accurate Biology, AG21102). Complementary DNA synthesis was carried out according to the manufacturer’s instructions using the Evo M-MLV RT pre-mix kit (Accurate Biology, AG11706). qRT-PCR was performed on a Rotor Gene 3000 real-time PCR system using 2× SYBR Green Pro Taq HS pre-mix (Accurate Biology, A3A2291). The reaction system was 20 μL, with the following conditions: pre-denaturation at 94 °C for 10 min, PCR amplification for 45 cycles with denaturation at 94 °C for 10 seconds, and annealing at 60 °C for 32 seconds. The list of primers used in the current study can be found in Supplementary Table 2.

### RNA FISH observation

RNA FISH detection was performed according to the manufacturer’s protocol using the FISH kit (Ribo Bio Technology, C10910). Cells were seeded in a 24-well plate. When cell density reached 50-60%, the culture medium was discarded and 4% paraformaldehyde was added. After washing with PBS, 500 μL of pre-cooled 0.3% Triton (Spark Jade, EA0011-A) was added to permeabilize the cells for 3 minutes, then PBS washed. 200 μL of prehybridization buffer was added to each well. After 30 minutes of incubation, 100 μL of 20 μM lnc668 was added to each well. RNA18S and RNU6 FISH probe mix were added to each well and incubated overnight. The cells were washed with SSC (Solarbio, S1030) and PBS solution, then stained with DAPI (DAPI: PBS = 1:400) for 10 minutes. Finally, the slides were sealed with anti-fluorescence quenching mounting agent and observed using confocal microscopy.

### RNA-binding protein immunoprecipitation (RIP) - PCR analysis

The RIP-PCR was performed following the instructions of the RNA Immunoprecipitation Kit (Genesee, Beijing, China). Cells were collected and lysed with lysis buffer containing 1% volume of protease inhibitor and 1% volume of RNase inhibitor. After centrifugation at 12,000 rpm for 10 minutes, the supernatant was collected. Simultaneously, Protein A + G beads were pretreated and then incubated with YTHDC1 and immunoglobulin G (IgG) antibodies for 3–4 hours. The Protein A + G beads conjugated with YTHDC1 and IgG antibodies were added to the cell lysate supernatant and incubated overnight. RNA was purified and extracted, followed by reverse transcription using the Evo M-MLV reverse transcription kit (Accurate, Changsha, China), and qPCR was performed. The qPCR conditions were as follows: an initial hold at 94°C for 600 seconds, followed by 45 cycles of amplification at 94°C for 5 seconds, 60°C for 32 seconds, and 72°C for 30 seconds.

### MeRIP-PCR analysis

MeRIP kit (BersinBio, Guangzhou, China) was used to detect the methylation modification in lnc668. Initially, RNA was extracted from 1 × 10^7^ cells following centrifugation at 1000 rpm at room temperature. Subsequently, the RNA samples were fragmented into approximately 100 nt-long fragments. Immunoprecipitation of methylated RNA was carried out using m^6^A antibody (abcam, ab208577) and normal rabbit IgG (Cell Signaling Technology, 2729) as a negative control. The RNA/antibody complexes were first incubated with Protein A/G magnetic beads (BersinBio, Guangzhou, China). This step facilitated the removal of non-specifically bound RNA and other impurities. Following this, the complexes underwent multiple washes to ensure thorough cleaning. RNA was then extracted from the washed RNA/antibody complexes. MERIP-PCR was used to analyze the purified RNA, allowing for the detection of immunoprecipitated RNA in each sample. The quantity of RNA isolated was calculated as a fraction of the initial input. This value was then normalized against the IgG control to determine the enrichment efficiency.

### Co-IP assay

The interaction between YTHDC1 and METTL3, as well as YTHDC1 and SRSF3, was detected using a Co-IP assay kit (Absin, China). MRC-5 cells were stimulated with TGF-β1 for 72 hours, harvested, and washed. They were subsequently lysed with pre-cooled lysis buffer, collected and thoroughly lysed using sonication. The lysates were centrifuged at 14,000 × *g* for 10 minutes at 4°C, and the supernatant (100 μL) was collected as the Input control. The negative control utilized normal rabbit IgG antibody (Cell Signal Technology, USA). Antibodies were pre-bound to magnetic beads to capture the antigens. Finally, the proteins bound to the magnetic beads were eluted and purified for Western blot analysis.

### CHIP- PCR analysis

According to the manufacturer’s instructions, chromatin immunoprecipitation (ChIP)- PCR analysis was performed using the SimpleChIP Enzymatic Chromatin IP Kit (Cell Signaling Technology, #9002, Danvers, MA, USA). First, cell samples were crosslinked with 1% formaldehyde (Macklin, F809702, Shanghai, China) at room temperature for 10 minutes. Subsequently, 2.5 M glycine (Meilunbio, MB4166, Dalian, China) was added to terminate the crosslinking for 5 minutes. Next, immunoprecipitation was carried out overnight using anti-Smad2/3 (Cell Signaling Technology, 8685, Danvers, MA, USA) or IgG (Cell Signaling Technology, 2729, Danvers, MA, USA). The antibody/antigen complexes were then recovered using Protein G Agarose beads (Cell Signaling Technology, 9007, Danvers, MA, USA) at 4°C for 2 hours. After two consecutive washings, 0.2 M NaCl was added to the elution buffer, and the crosslinking was reversed overnight at 65°C. Finally, the immunoprecipitated DNA was collected and subjected to PCR testing.

### Half-life of lnc668 analysis

1 × 10^6^ cells/mL of MRC-5 cells were seeded into six-well plates, including the following groups: Normal, TGF-β1 treatment group, TGF-β1 + si-METTL3 NC group, TGF-β1 + si-METTL3 group, METTL3 overexpression NC group, and METTL3 overexpression group. For the control group, cells were treated with 5 μg/mL streptomyces D (Aladdin, A13142-5 mg) alone for 4 hours. The TGF-β1-induced group cells were first treated with 5 ng/mL TGF-β1 for 24 hours, then co-treated with 5 μg/mL streptomyces D for 4 hours. In the si-METTL3 treatment group, cells were first treated with 5 ng/mL TGF-β1 for 24 hours, then co-treated with 10 μg/mL si-METTL3 for 24 hours, and finally co-treated with 5 μg/mL streptomyces D for 4 hours. In the METTL3 overexpression group, cells were first co-treated with 1 ng/mL overexpressed METTL3 for 48 hours, then co-treated with 5 μg/mL streptomyces D for 4 hours. qRT-PCR was employed to detect the levels of lnc668 at different time points of streptomyces D treatment, with GAPDH serving as the internal control.

### Immunofluorescence observation

MRC-5 cells were cultured in 24-well plates. The groups were divided as follows: normal group, TGF-β1 treated group, normal + 1,6-hexanediol group, and TGF-β1 + 1,6-hexanediol group. Cells were fixed with 4% paraformaldehyde for 30 minutes and permeabilized with 0.3% Triton X-100. Subsequently, cells were blocked with goat serum at room temperature for 1 hour and then incubated with anti-YTHDC1, METTL3, and SRSF3 antibodies at 4 °C for 12 hours. After that, fluorescently labeled secondary antibodies (Affinity, China) were incubated in the dark for 60 minutes. Each well was stained with 200 μL of DAPI (Solarbio, China) for 6 minutes. All images were obtained using a confocal laser scanning microscope. For fluorescence colocalization analysis, Image-Pro software was used to analyze the co-localization ratio of the two fluorescence signals (threshold Manders coefficient).

### FRAP analysis

Following transfection of live cells with plasmids overexpressing YTHDC1 tagged with green fluorescent protein (1 ng/mL) in 15 mm glass-bottom culture dishes (biosharp, BS-15-GJM, China) for 72 hours, droplet formation was observed under confocal microscopy. Photobleaching was performed within the defined ROI area, and the data were normalized to the frame with the highest average ROI intensity level. The bleached particles received 532 nm laser irradiation for 17.839 s at frame 0 post-bleaching. Each subsequent frame represented a recovery time of 10 seconds. Photobleaching was carried out along the selected ROC line, and the data were normalized to the frame with the highest average ROI intensity level. The bleached particles received 532 nm laser irradiation for 13 μs at frame 0 post-bleaching. Each subsequent frame represented recovery times at 4 seconds, 5 seconds, and 6.5 seconds, respectively.

### Nucleus-cytoplasm fractionation

First, 1 × 10^6^ MRC-5 cells were washed twice with PBS. The cell layer was scraped in 500 mL of PBS and centrifuged at 1050 rpm for 5 minutes. Nuclear and cytoplasmic RNA, as well as total RNA, were extracted from the cultured MRC-5 cells using the Paris Kit 50 RXNS (Life AM1921, USA) according to the manufacturer’s instructions. The enrichment of lnc668 was detected using the qRT-PCR method.

### RNA pull-down

The 3’ biotin ssRNA probes without m^6^A modification and the 3’ biotin ssRNA probes with m^6^A modification at site 555 were chemically synthesized and mixed with cell lysates for incubation. Biotin-binding protein magnetic beads were then added to each binding reaction, and the mixture was incubated at room temperature for 20 minutes. Finally, the beads were washed, and the recovered proteins were analyzed by protein gel electrophoresis.

### Agarose gel electrophoresis

1% agarose gel was prepared by weighing 1 g of agarose powder (AG, China) and added to 100 mL of 1× TAE buffer (SparkJade, China). The agarose powder and 1× TAE buffer were thoroughly mixed. The mixture was heated in a microwave until the agarose completely dissolved and the solution became clear. During heating, the solution was intermittently stirred to prevent overflow. After heating, the solution was allowed to cool to about 60°C, and an appropriate amount of DNA stain (ATGC, China) was added. The solution was then poured into a pre-prepared gel mold, the comb was inserted, and the gel was allowed to solidify completely, which took approximately 30 minutes. The solidified agarose gel was placed in an electrophoresis tank, and 1× TAE buffer was added until the gel surface was covered.

### Statistics and reproducibility

Statistical calculations and plotting were performed using GraphPad Prism 8. For in vitro experiments, the minimum sample size was three independent experimental data points, with data represented as the mean standard deviation (SD) of at least three independent repetitions. A t-test was used for comparisons between two groups, while one-way ANOVA was used for comparisons among three or more groups. The minimum sample size for in vivo experiments was six animals. Experimental data points were derived from the mean values of multiple technical replicates from independent experiments, with each data point representing one animal. All central values are presented as the mean, with error bars representing the standard error of the mean (SE). Sample sizes and p-values are shown on each graph, with p-values marked by an asterisk (*), where *p < 0.05. The sample size was chosen based on the biological and statistical significance of the group differences, and no data points were excluded. For cell experiments, all cells in each experiment were derived from the same batch of parental cells. The mice used in this study had the same genetic background (8-week-old C57BL/6 mice) and were sourced from the same batch. When conducting treatments such as nebulization, animals were randomly assigned to groups using a blinded method, and all animals were housed in the same environment and treated under the same protocols. For data collected using objective instruments (such as qPCR machines, microscope software, MicroCT measurements, and Western blotting), researchers did not apply blinding during data collection. However, in animal experiments, group assignments were made using a blinded randomization method. The variances between the groups undergoing statistical comparisons were similar.

### Reporting summary

Further information on research design is available in the Nature Portfolio Reporting Summary linked to this article.

## Supplementary information

Supplemental material

original data

original data

## Data Availability

The data supporting the findings of this study are available from the corresponding authors upon request.
