# Supplementary material for: YTHDC1 phase separation drives the nuclear export of m6A-modified lncNONMMUT062668.2 through the transport complex SRSF3–ALYREF–XPO5 to aggravate pulmonary fibrosis
Source: Cell Death Dis. 2025 Apr 12;16(1):279. doi: 10.1038/s41419-025-07608-x (PMC11993731; doi:10.1038/s41419-025-07608-x)
Supplement: Supplementary file 1 — Supplemental material [file 41419_2025_7608_MOESM1_ESM.docx]

**Supplementary figure legends**

**
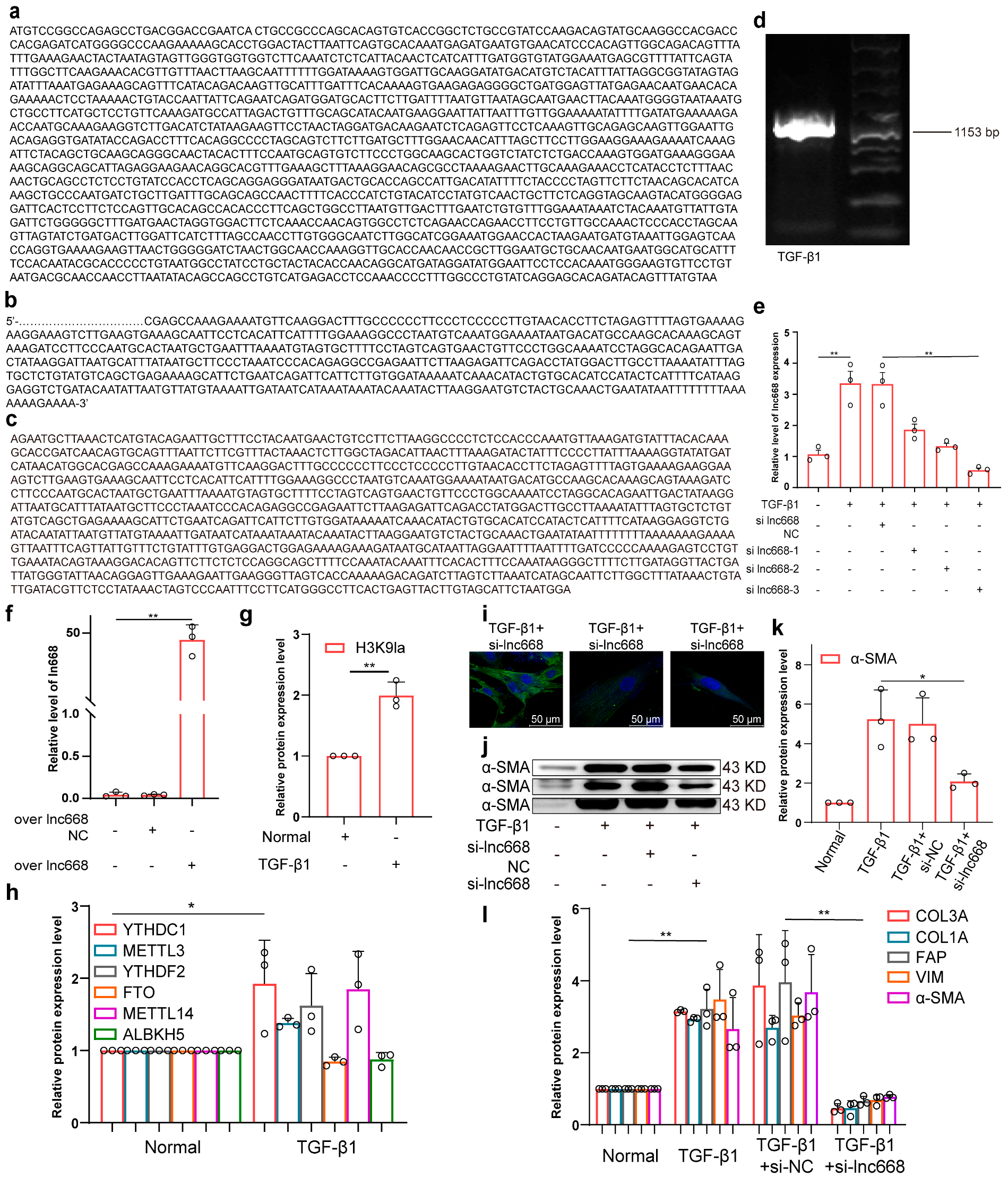
**

**Supplementary Fig. 1 |** **Full-length sequence of lnc668 and screening of the interference fragments of lnc668 and efficiency of lnc668 overexpression plasmids.** **a** Whole-transcriptome sequencing identified the full-length sequence of NONMMUT062668.2 in mice. **b** RACE obtained the partial lnc668 sequence in the human genome. **c** The full-length sequence of human lnc668 was obtained through PCR with specific primers and homology comparison. **d** Agarose gel electrophoresis revealed a highly expressed DNA band at 1153 bp in TGF-β1-stimulated MRC-5 cells, further validating the existence of lnc668 in the human genome. **e** Screening of the interference fragments of lnc668 in MRC-5 cells. qRT-PCR results demonstrated that si-lnc668 treatment significantly reduced lnc668 compared with TGF-β1 treatment and the third fragment was the most effective interference fragment. **f** Transfection of the lnc668 overexpression plasmid resulted in a significant increase in lnc668 expression in MRC-5 cells. **g** The expression differences of the bands were analyzed through grayscale values, with the data presented as the mean ± standard deviation. The asterisk indicates a significant upregulation of H3K9la protein in the TGF-β1 group (*p < 0.05). **h** The expression differences of the bands were analyzed through grayscale values, with the data presented as the mean ± standard deviation. The asterisk indicates a significant upregulation of YTHDC1 protein in the TGF-β1 group (*p < 0.05). **i** Three immunofluorescence analyses of α-SMA levels in the TGF-β1 + si-lnc668 group. **j** Three Western blot analyses of α-SMA levels in the TGF-β1 + si-lnc668 group. **k** The expression differences of the bands were analyzed based on grayscale values, and the data are presented as mean ± standard deviation. Asterisks indicate a significant decrease in α-SMA protein in the TGF-β1 + si-lnc668 group (*p < 0.05). **l** The expression differences of the bands were analyzed through grayscale values, with the data presented as the mean ± standard deviation. The asterisk indicates a significant upregulation of COL3A, COL1A, FAP, VIM and α-SMA proteins in the TGF-β1 treatment group. Significantly downregulated in the TGF-β1+si-lnc668 group. (*p < 0.05).


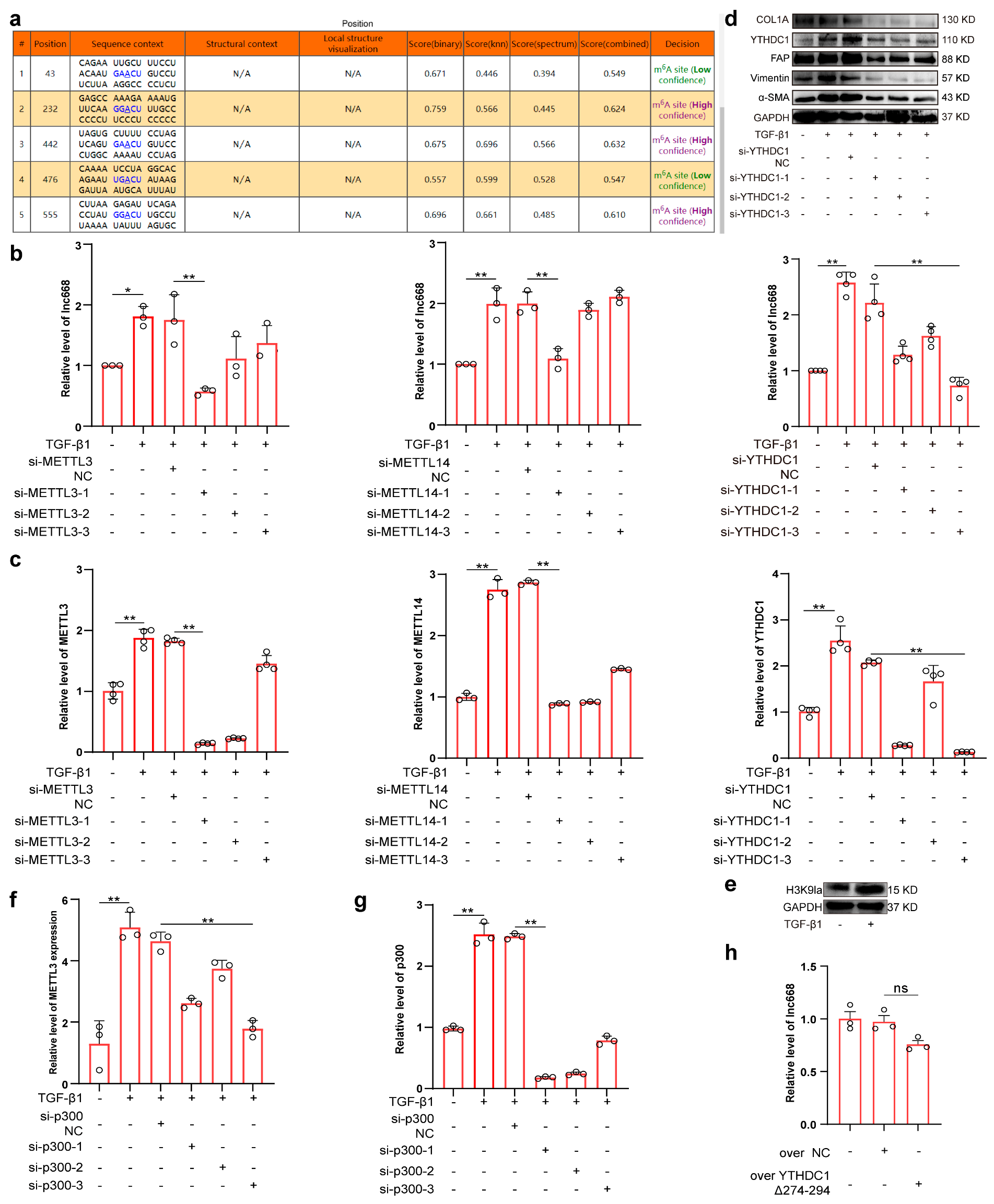


**Supplementary Fig. 2 | Prediction of the m^6^A sites of lnc668 and screening of interference fragments.** **a** SRAMP prediction (https://www.cuilab.cn/sramp) revealed that the high-confidence m^6^A sites of lnc668 were GGACU, GAACU, and UGACU, corresponding to positions 43, 232, 442, 476, and 555 of lnc668. **b** Three interference fragments were constructed for METTL3, METTL14, and YTHDC1, with qRT-PCR showing that the first METTL3 and METTL14 fragments effectively reduced the expression of lnc668, and the third YTHDC1 fragment also achieved a similar reduction in lnc668 expression. **c** The interference efficiency of each construct METTL3, METTL14, and YTHDC1 was assessed using the respective interference fragments. **d** Western blot analysis demonstrated that the three constructed YTHDC1 interference fragments effectively interfered with the high expression of YTHDC1 in TGF-β1-stimulated MRC-5 cells. The expression levels of proteins, such as FAP, VIM, α-SMA, and COL1A, in these cells significantly decreased compared with those in the control group. **e** H3K9la was markedly elevated in TGF-β1-stimulated cells. **f** Interference fragments of p300 were constructed, and qRT-PCR confirmed that the third fragment had the highest interference efficiency among fragments. **g** Three interference fragments of p300 were constructed to assess their interference efficiency. **h** qRT-PCR showed that the overexpression of YTHDC1-del-274-294 plasmid did not lead to an increase in lnc668 expression but instead resulted in a significant decrease in contrast to the overexpression of wild-type YTHDC1.


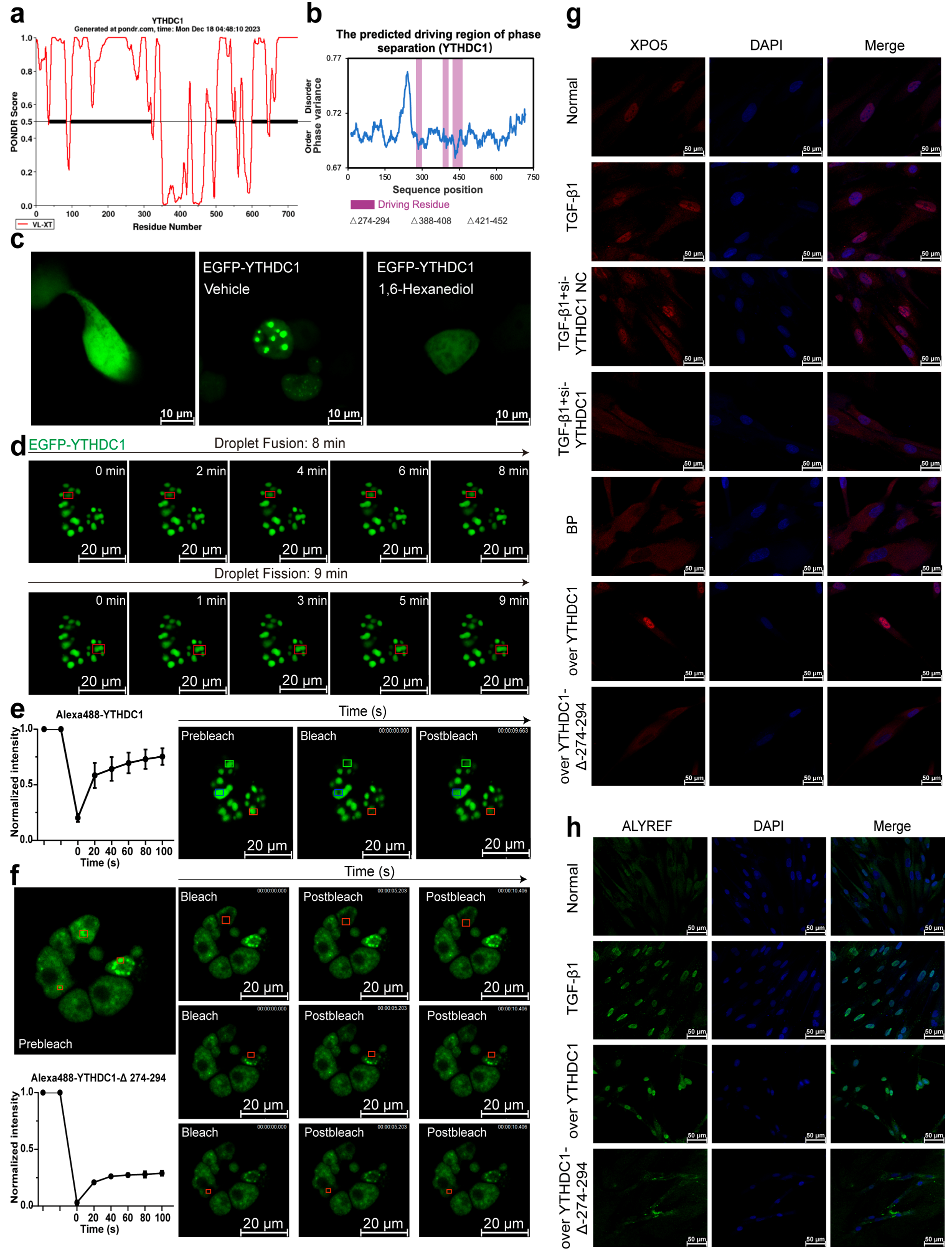


**Supplementary Fig. 3 | Phase separation ability of YTHDC1 in 293T cells.** **a** PONDR predictions indicated the presence of abundant IDRs within the YTHDC1 protein. Lines were drawn at positions 0 and 1 to indicate ordered or disordered regions to highlight reliable annotations. Regions with PONDR scores above 0.5 are considered disordered, whereas those with scores below 0.5 are considered ordered. **b** PSPHunter prediction revealed three IDRs in the YTHDC1 protein: amino acid fragments 274–294, 388–408, and 421–405. The overlapping IDR is amino acid fragment 274–294 from PONDR and PSPHunter predictions. **c** Nuclear punctate structures were not observed in the EGFP group, whereas overexpressed EGFP-YTHDC1 displayed numerous visible nuclear punctate structures, which were significantly reduced after the addition of 1,6-hexanediol. **d** Time-lapse live-cell images showed that EGFP-YTHDC1 nuclear droplets exhibited rapid phase separation dynamics. Two adjacent droplets fused at the 2 min time point and separated at the 3 min time point. **e** Overexpressing the EGFP-YTHDC1 plasmid, FRAP experiments showed rapid fluorescence recovery in the photobleached regions of the nuclear punctate structures. The fluorescence intensity in the bleached area recovered to 50% of its initial intensity within 17 s. **f** Overexpressing EGFP-YTHDC1-Δ274-294, FRAP experiments confirmed that nuclear puncta did not form. The fluorescence in the bleached area did not recover even after 100 s. **g** Immunofluorescence exhibited that XPO5 was expressed in the nuclei. After TGF-β1 stimulation, the nuclear expression of XPO5 significantly increased. Compared with TGF-β1 stimulation, si-YTHDC1 significantly reduced XPO5 expression in the nucleus and increased that in the cytoplasm. The overexpressed YTHDC1 promoted the nuclear XPO5 expression and decreased that in the cytoplasm. Under YTHDC1-Δ274-294 overexpression, the nuclear expression of XPO5 significantly decreased, with minimal expression in the cytoplasm. **h** Immunofluorescence revealed that ALYREF was expressed in the cytoplasm. After TGF-β1 stimulation, the nuclear expression of ALYREF significantly increased, whereas the cytoplasmic ALYREF expression decreased. Compared with TGF-β1 stimulation, YTHDC1 overexpression enhanced the nuclear ALYREF expression and weakened its cytoplasmic expression. YTHDC1-Δ274-294 overexpression significantly decreased nuclear ALYREF expression and increased cytoplasmic expression.

**Supplementary Tables**

**Supplementary Table1 | The product numbers and brands of antibodies.**

| Antibody Name | Item number | Brand of the antibody |
| --- | --- | --- |
| GAPDH | AF7021 | Affinity |
| α-SMA | AF1032 | Affinity |
| VIM | AF7013 | Affinity |
| COL1A | AF7001 | Affinity |
| COL3A | AF0136 | Affinity |
| FAP | Ab207178 | Abcam |
| Anti-N6-methyladenosine | Ab208577 | Abcam |
| YTHDC1 | 77422 | Cell Signaling TECHNOLOGY |
| METTL3 | OTI1B7 | Invitrogen |
| SRSF3 | 33-4200 | Invitrogen |
| PICALM | 67564-1-Ig | Proteintech |
| METTL14  YTHDF2  FTO  ALBKH5  XPO5  ALYREF  lamin B1  β-tubulin  MYC tag | 26158-1-AP  24744-1-AP  27226-1-AP  49015  PAB53127  RMAB48942  RMAB60267  RMAB60262  60003-2-Ig | Proteintech  Proteintech  Proteintech  Cell Signaling TECHNOLOGY  Bioswamp  Bioswamp  Bioswamp  Bioswamp  Proteintech |

**Supplementary Table2 | List of primers used in the current study.**

| **Primer_name** | **Sequence (5'-3')** |
| --- | --- |
| lnc668-qPCR-For | CTTTTCCTAGTCAGTGAACTGTTCC |
| lnc668-qPCR-Rev  lnc668-PCR-For  lnc668-PCR-Rev | TCTCAGCTGACATACAGAGCACTAA  AGAATGCTTAAACTCATGTACAGAATTG  TCCATTAGAATGCTACAAGTAACTCAG |
| GAPDH-qPCR-For | TGCACCACCAACTGCTTAGC |
| GAPDH-qPCR-Rev | GGCATGGACTGTGGTCATGAG |
| U6-qPCR-For | CTCGCTTCGGCAGCACA |
| U6-qPCR-Rev | AACGCTTCACGAATTTGCGT |
| PICALM-qPCR-For1 | AGCCCCACTTCCTACCAGATG |
| PICALM-qPCR-Rev1  METTL3-qPCR-For  METTL3-qPCR-Rev  METTL14-qPCR-For  METTL14-qPCR-Rev  YTHDC1-qPCR-For  YTHDC1-qPCR-Rev  p300-qPCR-For  p300-qPCR-Rev | TGAGAACTGTTATTTCCCCATGC  AGATGGGGTAGAAAGCCTCCT  TGGTCAGCATAGGTTACAAGAGT  GTTGGAACATGGATAGCCGC  CAATGCTGTCGGCACTTTCA  AACTGGTTTCTAAGCCACTGAGC  GGAGGCACTACTTGATAGACGA  TTGTGAAGAGCCCCATGGAT  GCTTTGCATCACTGGGTCAA |
