# Supplementary material for: YTHDC1 phase separation drives the nuclear export of m6A-modified lncNONMMUT062668.2 through the transport complex SRSF3–ALYREF–XPO5 to aggravate pulmonary fibrosis
Source: Cell Death Dis. 2025 Apr 12;16(1):279. doi: 10.1038/s41419-025-07608-x (PMC11993731; doi:10.1038/s41419-025-07608-x)
Supplement: Supplementary file 2 — original data [file 41419_2025_7608_MOESM2_ESM.pdf]

Fig. 1a

	Normal	24h	48h	72h
	1.0796701	1.35321816	1.67039441	2.50392166
	1.11204242	1.32204624	1.65945353	2.47308356
	0.96875112	1.05145759	1.86430468	2.39411737
	0.83953636	0.86772804	1.93608511	3.81688868

fig. 1c

		nucleus			cytoplas		
Normal	lncUTPF	0.838849	0.83791	0.745429	0.16115	0.16209	0.25457
	U6	0.911248	0.914555	0.901245	0.08875	0.08545	0.09876
	GAPDH	0.239112	0.22673	0.201111	0.76089	0.77327	0.79889
TGF-β1	lncUTPF	0.267947	0.277571	0.2748	0.73205	0.72243	0.7252
	U6	0.912916	0.911807	0.903686	0.08708	0.08819	0.09631
	GAPDH	0.115286	0.100621	0.098755	0.88471	0.89938	0.90125
TGF-β 1+si-NC	lncUTPF	0.385941	0.34891	0.360012	0.61406	0.65109	0.63999
	U6	0.897482	0.84164	0.901861	0.10252	0.15836	0.09814
	GAPDH	0.084905	0.065697	0.066123	0.9151	0.9343	0.93388
TGF-β 1+si-lnc668	lncUTPF	0.387584	0.333333	0.360012	0.61242	0.66667	0.63999
	U6	0.917749	0.931262	0.922337	0.08225	0.06874	0.07766
	GAPDH	0.098139	0.099373	0.085445	0.90186	0.90063	0.91456
over lnc668 NC	lncUTPF	0.722429	0.715426	0.72658	0.27757	0.28457	0.27342
	U6	0.849779	0.860091	0.860091	0.15022	0.13991	0.13991
	GAPDH	0.197791	0.18181	0.157438	0.80221	0.81819	0.84256
over lnc668	lncUTPF	0.479218	0.489604	0.463674	0.52078	0.5104	0.53633
	U6	0.891598	0.891598	0.877454	0.1084	0.1084	0.12255
	GAPDH	0.15836	0.169763	0.174704	0.84164	0.83024	0.8253

Fig. 2a

MERIP	IgG	MERIP	IgG
1.573	0.68	5.517	0.191
0.962	0.272	4.45	0.154
1.031	0.22	4.419	0.161
Normal		TGF-β1	

Fig. 2b

MERIP	IgG	MERIP	IgG	MERIP	IgG
0.08	0.067	0.969	0.186	44.135	1.746
0.073	0.077	1.168	0.199	26.062	0.755
0.191	0.09	0.311	0.036	42.337	1.342
Normal		over lnc668 NC		over lnc668-WT	

MERIP	IgG	MERIP	IgG	MERIP	IgG	MERIP	IgG
1.64	0.072	7.381	0.714	8.362	0.186	1.782	0.969
2.32	0.052	8.077	0.685	7.802	0.195	1.77	0.648
2.005	0.122	7.966	0.596	7.589	0.177	1.782	0.803
over lnc668-Mut 3		over lnc668-Mut 232		over lnc668-Mut 442		over lnc668-Mut 555	

Fig. 2d

MERIP	IgG	MERIP	IgG
1.573	0.68	5.517	0.191
0.962	0.272	4.45	0.154
1.031	0.22	4.419	0.161
Normal		TGF-β1	

MERIP	IgG	MERIP	IgG	MERIP	IgG
4.45	0.512	0.169	0.144	0.364	0.047
3.928	0.208	0.572	0.246	0.237	0.038
4.299	0.136	0.244	0.206	0.526	0.039
si-NC		si-METTL3		si-METTL14	

Fig. 2e

1	1.981601	2.178497	0.587774
1	2.1386	1.650992	0.50581
1	1.79005	2.445281	0.619854
Normal	TGF-β1	TGF-β1+si-NC	TGF-β1+si-METTL3
1	2.317267	3.231993	0.507832
1	2.717771	1.935121	0.802417
1	2.553408	1.684621	0.76973
1	2.717771	2.017298	0.842311
Normal	TGF-β1	TGF-β1+si-NC	TGF-β1+si-YTHDC1
1	1.725	1.852	1.25
1	2	1.923	0.92
1	2.25	2.215	1.1
Normal	TGF-β1	TGF-β1+si-NC	TGF-β1+si-METTL14
1	1.290078	2.598102	
1	1.421543	2.47505	
1	1.48191	2.325366	
1	1.738036	1.750125	
Normal	over METTL3 NC	over METTL3	
0.868587	0.963757	2.123938	
1.025794	1.178328	3.775694	
1.114767	1.130328	3.264228	
0.990852	1.391597	2.802553	
Normal	over YTHDC1 NC	over YTHDC1	
0.777367	1.138132	6.007096	
0.937355	1.21982	7.045333	
1.10701	1.194716	7.094337	
1.178268	1.316464	5.566104	

	Normal	over METTL14 NC	over METTL14	
Fig. 2f				
	IgG	H3K9la	IgG	H3K9la
	1.04972	1.3566	1.0401	4.6161
	1.11729	1.30134	1.1225	3.9632
	0.85263	1.31039	0.8566	3.2868

	Normal		TGF-β1	
Fig. 2g				
	1.588269	5.657895	4.296875	2.055031
	1.852979	4.760158	4.785938	1.510764
	0.437823	4.848222	4.827586	1.78488
	Normal	TGF-β1	TGF-β1+si-NC	TGF-β1+si-p300

Fig. 2h							
	Time (h)	Normal	TGF-β1	TGF-β1+si-METTL3 NC	TGF-β 1+si-METTL3	over METTL3 NC	over METTL3
	0	1	1	1	1	1	1
	2	0.882703	0.952944	0.950147	0.80664	0.85264	0.93735
	4	0.806642	0.884662	0.946802	0.75611	0.81979	0.87256
	6	0.790041	0.884647	0.883044	0.61985	0.80478	0.772
	8	0.747425	0.816546	0.869943	0.56383	0.77022	0.74055
	Time (h)	Normal	TGF-β1	TGF-β1+si-YTHDC1 NC	TGF-β 1+si-YTHDC1	over YTHDC1 NC	over YTHDC1
	0	1	1	1	1	1	1
	2	0.849277	0.965742	0.902896	0.7421	0.909	0.90216
	4	0.445376	0.770759	0.852933	0.4338	0.77559	0.87465
	6	0.423252	0.634824	0.717712	0.39303	0.71116	0.84054
	8	0.416958	0.611834	0.690846	0.31016	0.65951	0.75222

Fig. 2k					
	0.967777	0.928354	2.20801	1.387749	0.74368
	1.037238	0.954453	1.756553	1.793461	0.74368
	0.994985	1.142938	2.223368	2.833817	0.99499
	Normal	over METTL3 NC	over METTL3	over METTL3+si-YTHDC1 NC	over METTL3 +si-YTHDC1

Fig. 2l						
	YTHDC1 Ab	IgG	YTHDC1 Ab	IgG	YTHDC1 Ab	IgG
	0.8566	0.8448	9.12611	1.9053	8.359	0.7287
	1.2368	1.3819	11.0809	0.7371	13.865	1.0449
	0.7774	0.8566	10.4107	0.712	6.8844	1.3134

	Normal		TGF-β1		TGF-β1+over NC	
	YTHDC1 Ab	IgG	YTHDC1 Ab	IgG		
	34.776	1.1408	8.5374	1.2687		
	33.359	1.057	9.2467	1.4777		
	29.651	0.8293	9.3898	0.5334		
	TGF-β1+over lnv668-WT		TGF-β1+over lnc668-Mut 555			

Fig. 2m	YTHDC1 Ab	IgG	YTHDC1 Ab	IgG	YTHDC1 Ab	IgG
	1.11214	0.8085	8.0371	0.8448	7.078	0.6846
	1.85746	1.2426	8.2059	1.0401	8.8356	1.234
	0.70385	0.9954	9.9636	1.1381	9.4698	1.1837
	Normal		TGF-β1		TGF-β1+si-NC	
	YTHDC1 Ab	IgG	YTHDC1 Ab	IgG	YTHDC1 Ab	IgG
	1.7331	0.9096	1.7613	1.0693	1.879	0.8351
	2.1485	0.9287	1.5874	0.9772	2.8284	0.9138
	2.3674	1.1837	1.1865	0.9571	2.8879	1.3104
	TGF-β1+si-METTL3		TGF-β1+si-METTL3+over NC		TGF-β1+si-METTL3+over lnc668	

Fig. 3b			nucleus			cytoplasm	
Normal	lncUTPF	0.963929	0.975743	0.996241	0.03607	0.02426	0.00376
	U6	0.931262	0.975578	0.945971	0.06874	0.02442	0.05403
	GAPDH	0.26659	0.19235	0.183882	0.73341	0.80765	0.81612
TGF-β1	lncUTPF	0.38266	0.446487	0.472239	0.61734	0.55351	0.52776
	U6	0.963929	0.968238	0.847105	0.03607	0.03176	0.1529
	GAPDH	0.107734	0.151999	0.152895	0.89227	0.848	0.84711
TGF-β 1+si-NC	lncUTPF	0.618976	0.647934	0.384299	0.38102	0.35207	0.6157
	U6	0.907245	0.947716	0.943066	0.09276	0.05228	0.05693
	GAPDH	0.196694	0.188079	0.137425	0.80331	0.81192	0.86258
TGF-β 1+si-METTL3	lncUTPF	0.646351	0.692319	0.750654	0.35365	0.30768	0.24935
	U6	0.772053	0.704003	0.66203	0.22795	0.296	0.33797
	GAPDH	0.26659	0.300348	0.288826	0.73341	0.69965	0.71117
TGF-β 1+si-YTHDC	lncUTPF	0.655799	0.687872	0.635183	0.3442	0.31213	0.36482
	U6	0.893591	0.935985	0.929919	0.10641	0.06402	0.07008
	GAPDH	0.198893	0.206737	0.153795	0.80111	0.79326	0.84621

Fig. 3c

		nucleus			cytoplasm		
Normal	lncUTPF	0.757085	0.929465	0.949072	0.24292	0.07054	0.05093
	U6	0.73341	0.859255	0.835066	0.26659	0.14075	0.16493
	GAPDH	0.017632	0.007337	0.001794	0.98237	0.99266	0.99821
over METTL3 NC	lncUTPF	0.966263	0.956249	0.985922	0.03374	0.04375	0.01408
	U6	0.859255	0.83791	0.81819	0.14075	0.16209	0.18181
	GAPDH	0.000214	0.008833	0.092755	0.99979	0.99117	0.90725
over METTL3	lncUTPF	0.285987	0.27342	0.352066	0.71401	0.72658	0.64793
	U6	0.846205	0.763401	0.789832	0.1538	0.2366	0.21017
	GAPDH	0.118868	0.131774	0.075216	0.88113	0.86823	0.92478
over METTL3+si-YTHDC1 NC	lncUTPF	0.439645	0.47403	0.427729	0.56036	0.52597	0.57227
	U6	0.785192	0.796652	0.816118	0.21481	0.20335	0.18388
	GAPDH	0.33797	0.307681	0.2	0.66203	0.69232	0.8
over METTL3+si-YTHDC1	lncUTPF	0.652663	0.825296	0.663579	0.34734	0.1747	0.33642
	U6	0.821262	0.84164	0.893591	0.17874	0.15836	0.10641
	GAPDH	0.167818	0.11458	0.180782	0.83218	0.88542	0.81922

Fig. 4f

1.032588	5.881828	4.396223	1.032588	1.03977	2.52497	1.01837
0.976888	5.043364	4.101821	1.280107	1.1067	3.08713	1.21948
0.990524	5.850762	4.614789	1.470457	0.91781	3.3087	1.05429
Normal	TGF-β1	TGF-β1+si-NC	TGF-β1+si-YTHDC1	TGF-β1+si-YTHDC1+over NC	TGF-β1+si-YTHDC1+over YTHDC1	TGF-β1+si-YTHDC1+over YTHDC1-Δ274-294

Fig. 4h

		nucleus			cytoplasm		
Normal	lncUTPF	0.922337	0.883293	0.886121	0.07766	0.11671	0.11388
	U6	0.600838	0.573967	0.589146	0.39916	0.42603	0.41085
	GAPDH	0.057683	0.048313	0.046436	0.94232	0.95169	0.95356
TGF-β1	lncUTPF	0.092173	0.105751	0.100621	0.90783	0.89425	0.89938
	U6	0.801107	0.809795	0.788679	0.19889	0.19021	0.21132
	GAPDH	0.025779	0.032626	0.025779	0.97422	0.96737	0.97422
TGF-β1+si-YTHDC1 NC	lncUTPF	0.198893	0.239112	0.212479	0.80111	0.76089	0.78752
	U6	0.917749	0.913465	0.911248	0.08225	0.08654	0.08875
	GAPDH	0.011703	0.010134	0.01528	0.9883	0.98987	0.98472
TGF-β1+si-YTHDC	lncUTPF	0.556937	0.698193	0.69673	0.44306	0.30181	0.30327
	U6	0.764651	0.797773	0.736112	0.23535	0.20223	0.26389

YTHDC 1	GAPDH	0.089314	0.102518	0.101882	0.91069	0.89748	0.89812
TGF-β 1+over NC	lncUTPF	0.723817	0.680383	0.672799	0.27618	0.31962	0.3272
	U6	0.77327	0.788679	0.89293	0.22673	0.21132	0.10707
	GAPDH	0.061568	0.054384	0.0404	0.93843	0.94562	0.9596
TGF-β 1+over YTHDC	lncUTPF	0.082775	0.09102	0.088752	0.91723	0.90898	0.91125
	U6	0.723817	0.751949	0.754526	0.27618	0.24805	0.24547
	GAPDH	0.399162	0.278963	0.253258	0.60084	0.72104	0.74674
TGF-β 1+over YTHDC	lncUTPF	0.96195	0.979523	0.983863	0.03805	0.02048	0.01614
	U6	0.916166	0.908405	0.912363	0.08383	0.0916	0.08764
	GAPDH	0.015176	0.021184	0.011155	0.98482	0.97882	0.98885

Fig. 7c

	1	3.099867	2.120421	1.269787
	1	3.566334	2.478602	1.321683
	1	3.002338	2.44583	1.307093
	Normal	TGF-β1	TGF-β1+si-NC	TGF-β1+si-lnc668

Fig. 7d

	Time(h)	Normal	TGF-β1	TGF-β1+si-lnc668 NC	TGF-β 1+si-lnc668
	0	1	1	1	1
	2	0.92	0.89	0.824	0.648
	4	0.83	0.82	0.641	0.59
	6	0.544	0.53	0.456	0.421
	8	0.48	0.46	0.363	0.295

Supplementary Fig. 1e

	1	3.460547	3.429436	2.183858	1.2	0.67
	1.32	3.960547	3.929436	1.578324	1.3	0.58
	0.89	2.646055	2.629436	1.83456	1.5	0.435
	Normal	TGF-β1	TGF-β1+si-NC	TGF-β1+si-lnc668-1	TGF-β 1+si-lnc668-2	TGF-β 1+si-lnc668-3

Supplementary Fig. 1f

	0.029564	0.050067	47.83518
	0.025916	0.032577	36.25228
	0.07911	0.043586	53.81737
	Normal	over lnc668 NC	over lnc668

Supplementary Fig. 2b						
	1	1.981601	2.178497	0.587774	1.52274	1.70133
	1	1.650992	1.347234	0.50581	0.82932	1.16205
	1	1.79005	1.729074	0.619854	0.98623	1.24401
	Normal	TGF-β1	TGF-β1+si-NC	TGF-β1+si-METTL3-1	TGF-β1+si-METTL3-2	TGF-β1+si-METTL3-3
	1	1.725	1.852	1.25	1.78	2.22
	1	2	1.923	0.92	1.89	2.1
	1	2.25	2.215	1.1	2	2.01
	Normal	TGF-β1	TGF-β1+si-NC	TGF-β1+si-METTL14-1	TGF-β1+si-METTL14-2	TGF-β1+si-METTL14-3
	1	2.317267	2.231993	1.166692	1.42645	0.50783
	1	2.717771	1.935121	1.259127	1.70814	0.80242
	1	2.553408	2.684621	1.207836	1.55017	0.76973
	1	2.717771	2.017298	1.507778	1.80553	0.84231
	Normal	TGF-β1	TGF-β1+si-NC	TGF-β1+si-YTHDC1-1	TGF-β1+si-YTHDC1-2	TGF-β1+si-YTHDC1-3

Supplementary Fig. 2c						
	0.95429	1.818186	1.780769	0.156312	0.23857	1.36841
	0.842355	1.98963	1.843567	0.152038	0.22885	1.36841
	1.119225	1.708227	1.793155	0.140876	0.21651	1.43644
	1.111494	2.003469	1.882304	0.121793	0.22106	1.65004
	Normal	TGF-β1	TGF-β1+si-NC	TGF-β1+si-METTL3-1	TGF-β1+si-METTL3-2	TGF-β1+si-METTL3-3
	0.981686	2.630774	2.901874	0.895242	0.90958	1.46856
	0.954842	2.689265	2.868014	0.864178	0.92442	1.44373
	1.066832	2.935969	2.829343	0.8966	0.91771	1.44373
	Normal	TGF-β1	TGF-β1+si-NC	TGF-β1+si-METTL14-1	TGF-β1+si-METTL14-2	TGF-β1+si-METTL14-3
	1.03766	2.334856	2.118926	0.268563	1.79419	0.14096
	1.089249	2.502436	2.004626	0.266708	1.15136	0.12792
	0.884745	2.367449	2.118926	0.295931	1.83189	0.13061
	1.052145	3.017457	2.060984	0.285851	1.89649	0.1371
	Normal	TGF-β1	TGF-β1+si-NC	TGF-β1+si-YTHDC1-1	TGF-β1+si-YTHDC1-2	TGF-β1+si-YTHDC1-3

Supplementary Fig. 2f						
	1.588269	5.657895	4.296875	2.596992	3.94137	2.05503
	1.852979	4.760158	4.785938	2.787055	3.8549	1.51076
	0.437823	4.848222	4.827586	2.468629	3.42874	1.78488
	Normal	TGF-β1	TGF-β1+si-NC	TGF-β1+si-p300-1	TGF-β1+si-p300-1	TGF-β1+si-p300-1

Supplementary Fig. 2g						
	1.026334	2.374296	2.481953	0.195806	0.22492	0.86304
	0.957603	2.458026	2.441429	0.186533	0.275	0.72072
	0.957603	2.72735	2.534214	0.163516	0.2394	0.77782
	Normal	TGF-β1	TGF-β1+si-NC	TGF-β1+si-p300-1	TGF-β1+si-p300-1	TGF-β1+si-p300-1

Supplementary Fig. 2h			
	0.959371	1.086855	0.732124
	0.90762	0.926691	0.829412
	1.13301	0.90762	0.712104
	Normal	over NC	over YTHDC1-Δ 274-294
