# Supplementary material for: YTHDC1 phase separation drives the nuclear export of m6A-modified lncNONMMUT062668.2 through the transport complex SRSF3–ALYREF–XPO5 to aggravate pulmonary fibrosis
Source: Cell Death Dis. 2025 Apr 12;16(1):279. doi: 10.1038/s41419-025-07608-x (PMC11993731; doi:10.1038/s41419-025-07608-x)
Supplement: Supplementary file 3 — original data [file 41419_2025_7608_MOESM3_ESM.docx]

Fig. 1e

COL3A COL1A







FAP VIM




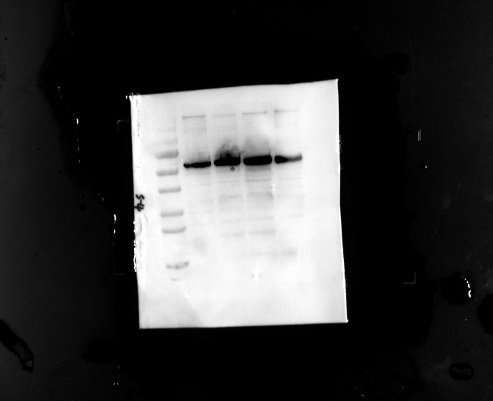


α-SMA GAPDH


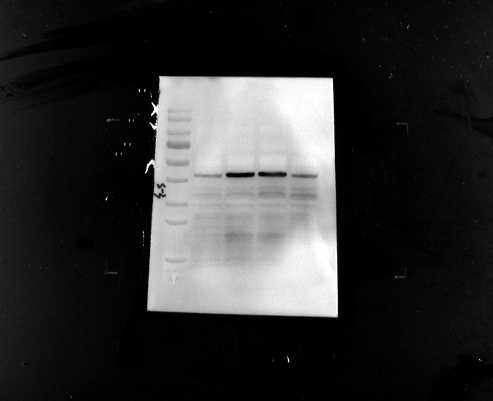




COL3A COL1A







FAP VIM







α-SMA GAPDH







Fig. 2c

YTHDC1 METTL3







YTHDF2 FTO







METTL14 ALBKH5







GAPDH





Fig. 2i

IB: YTHDC1 IB: METTL3







YTHDC1-Input METTL3-Input







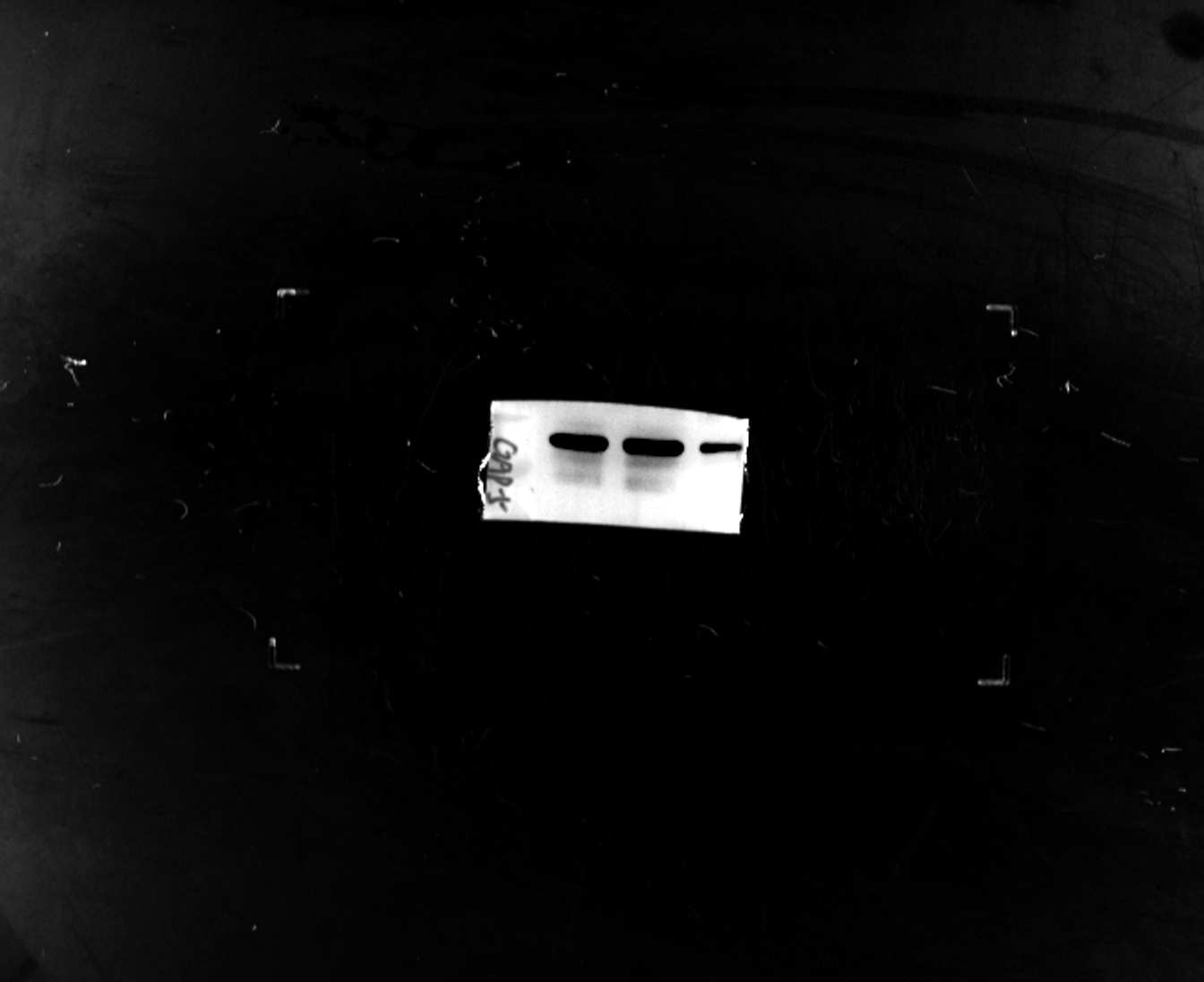

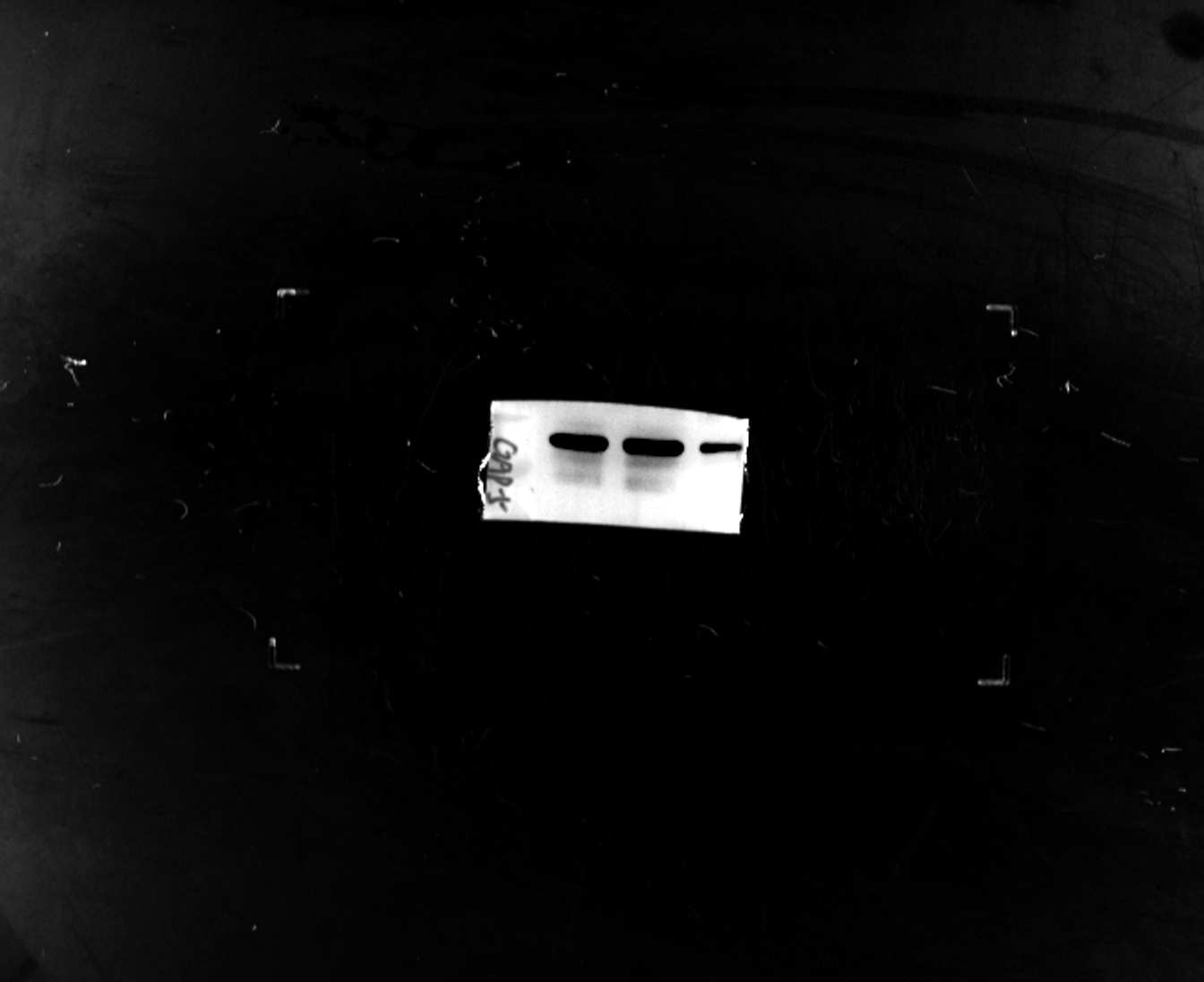


GAPDH





Fig. 4g

Myc-YTHDC1-Δ274-294 Myc-YTHDC1 WT







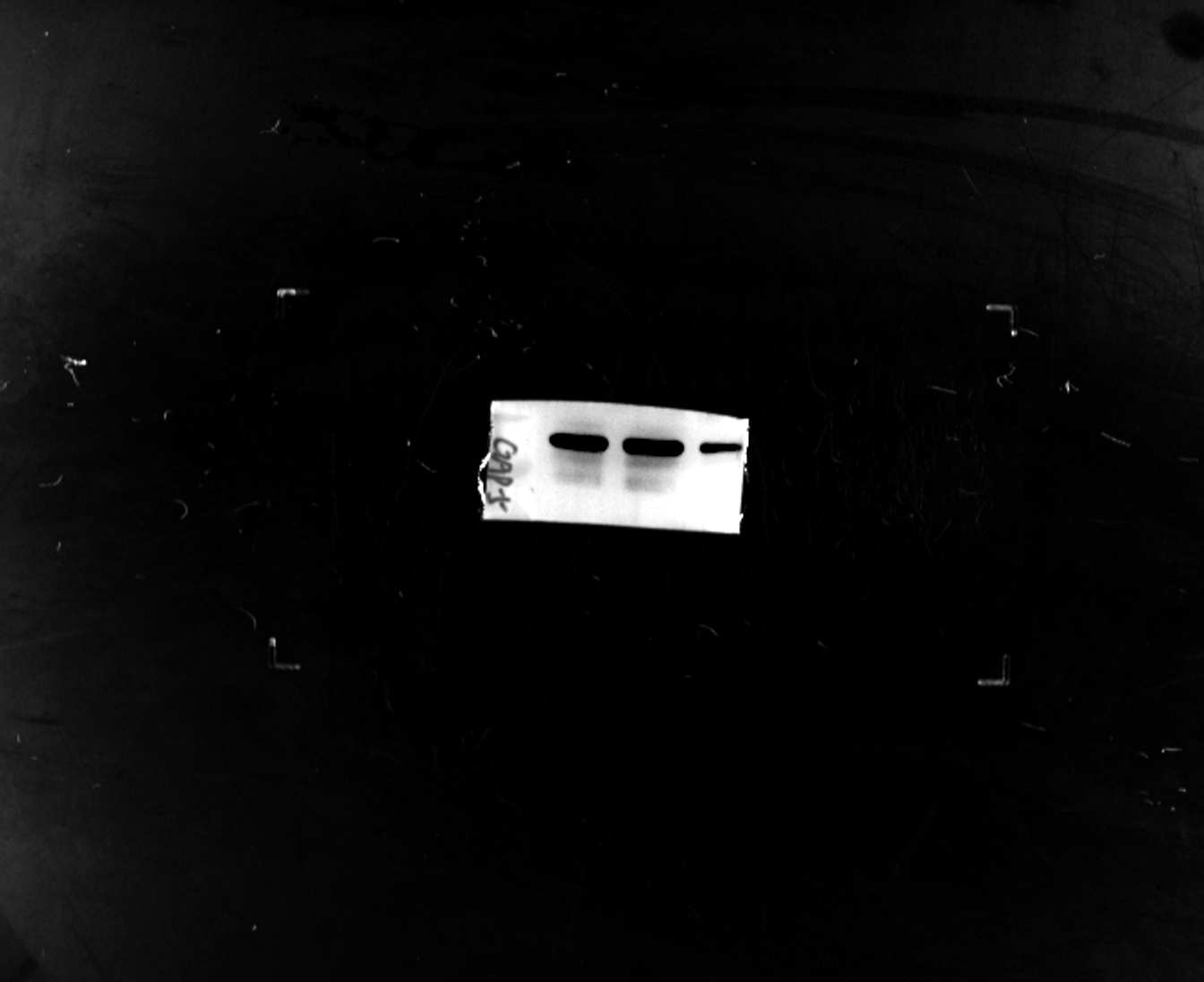

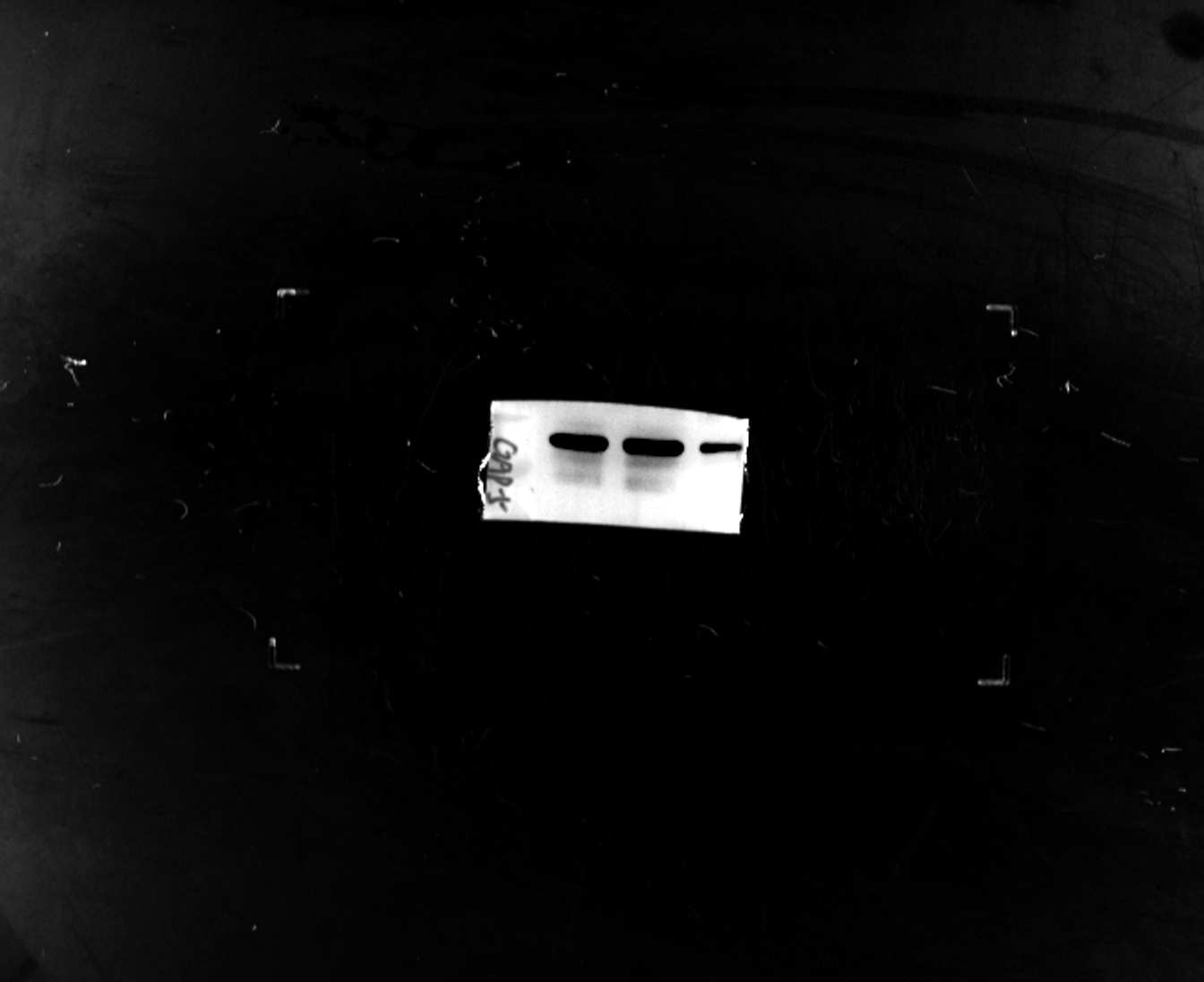


GAPDH





Fig. 8a

IB: YTHDC1 IB: SRSF3


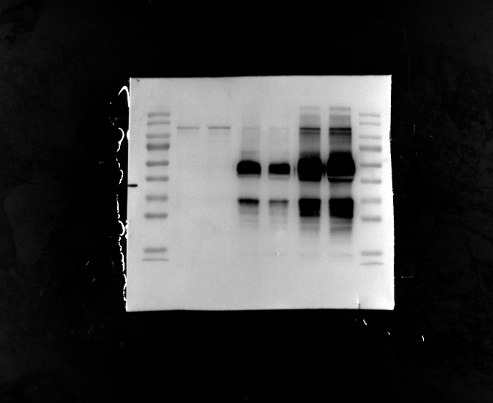




YTHDC1-Input SRSF3-Input







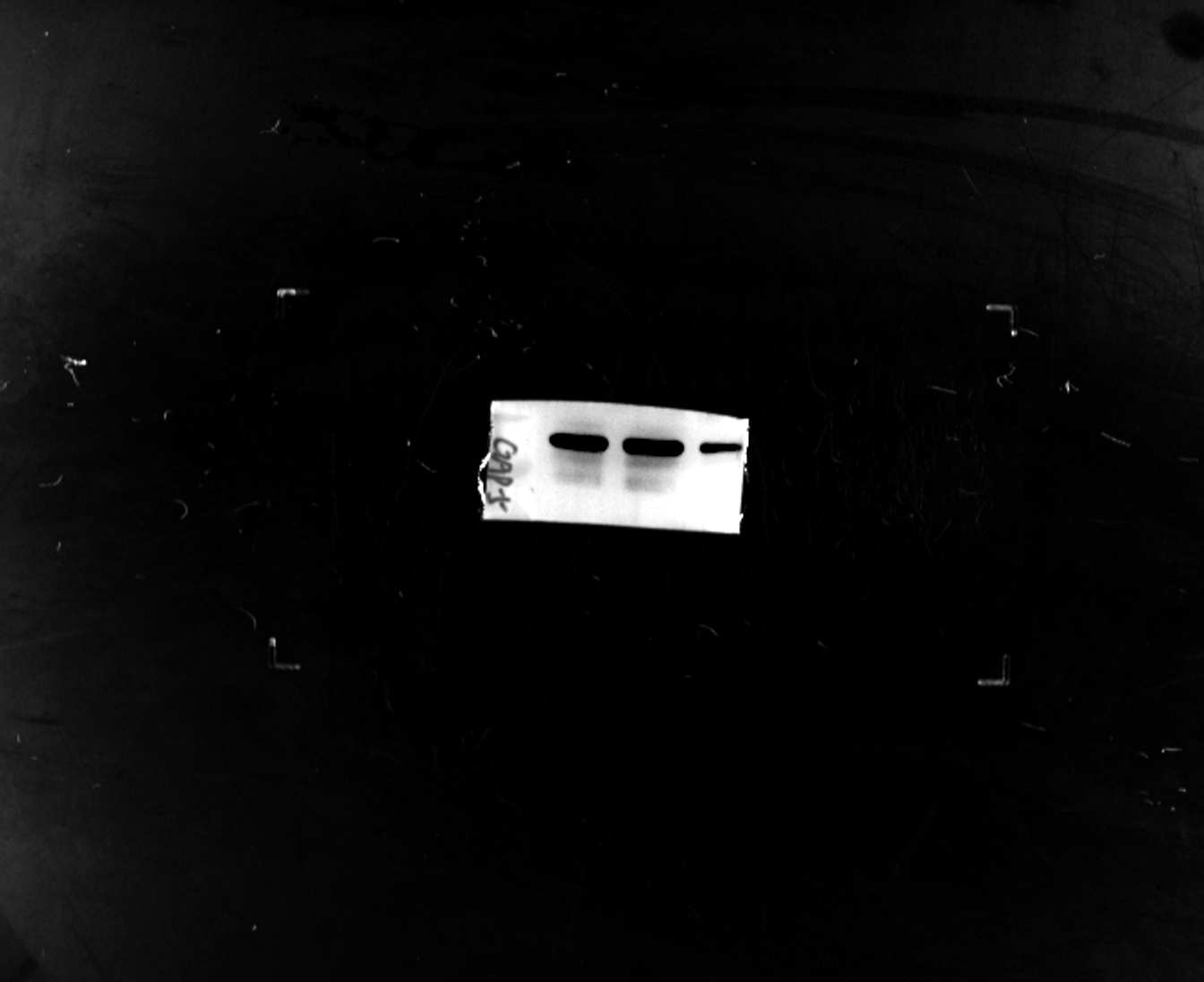

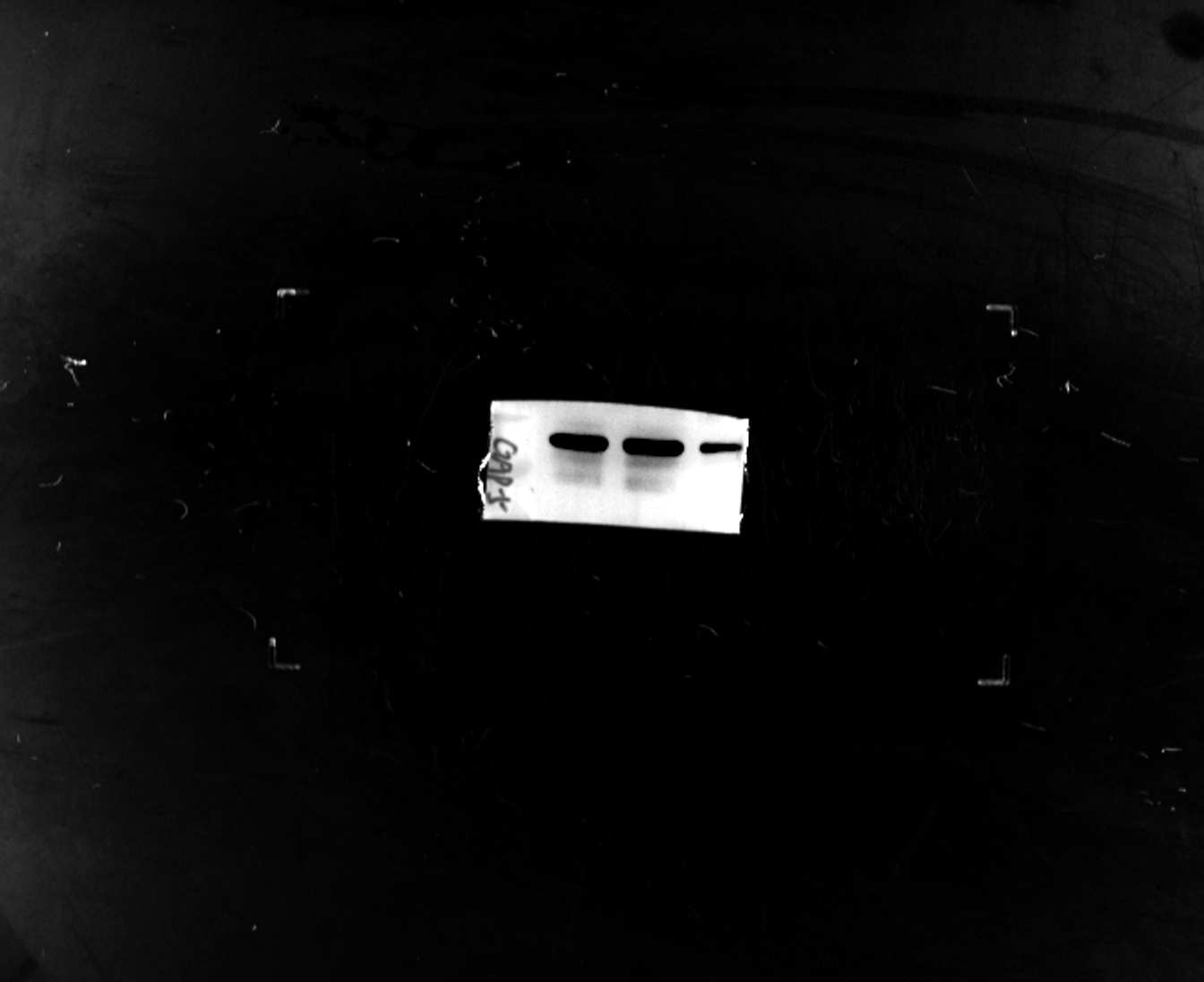


GAPDH





Fig. 8e

IB: Myc IB: SRSF3




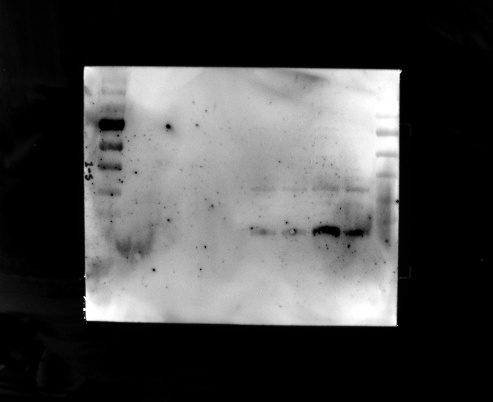


Myc-Input Myc-Input


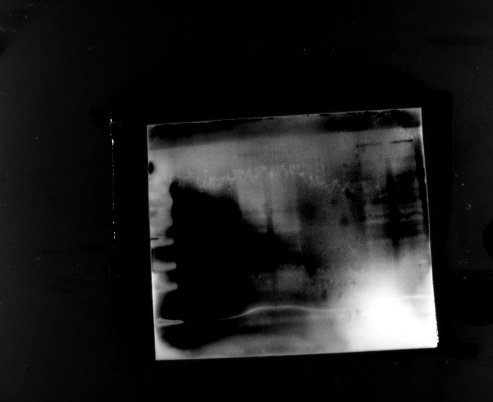

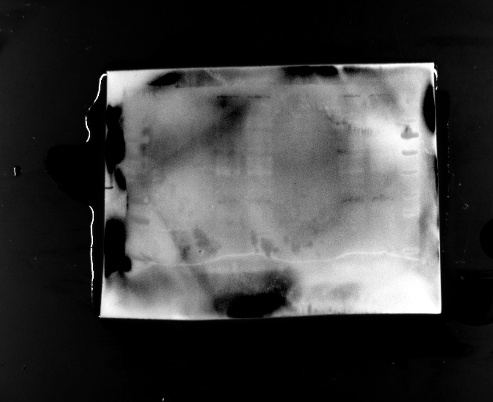


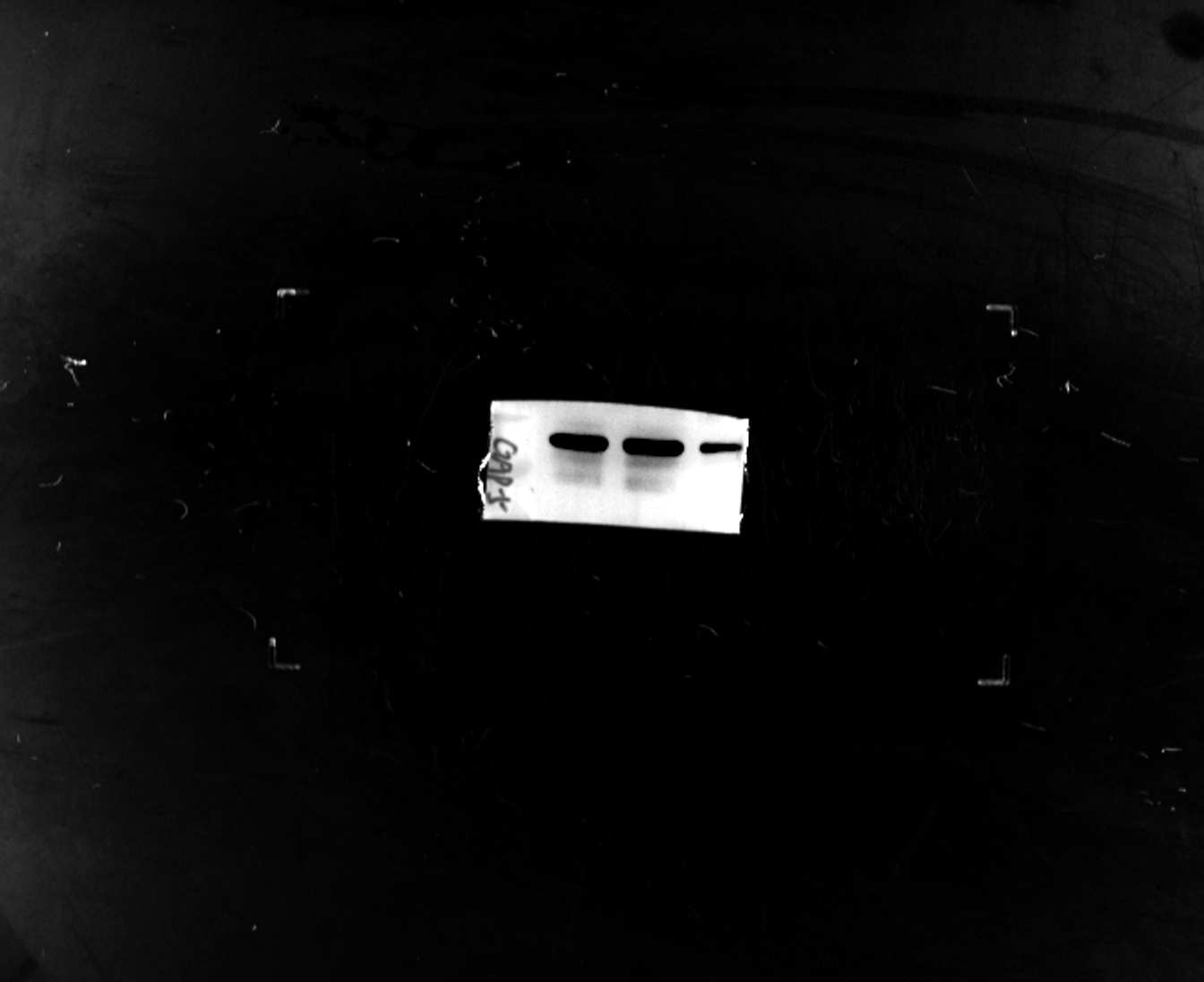

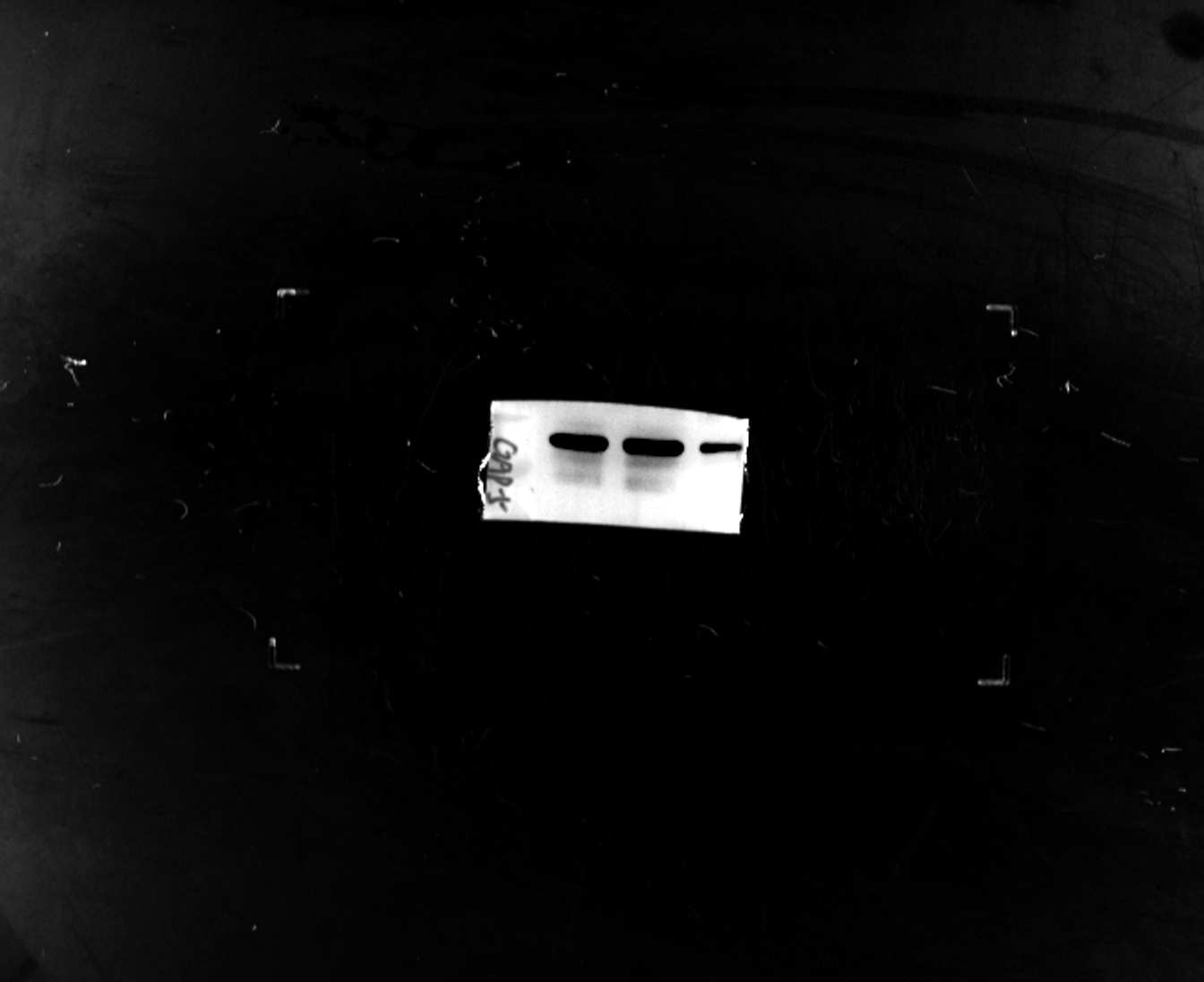


SRSF3-Input GAPDH




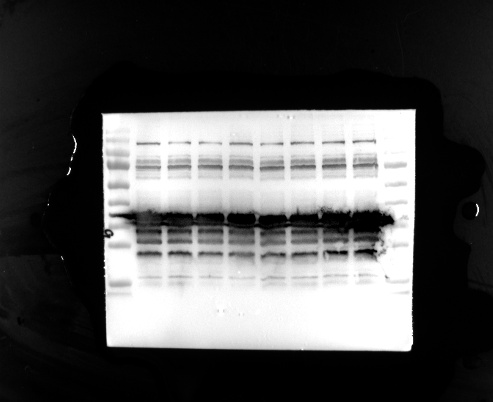


Fig. 8f

XPO5 Lamin B1




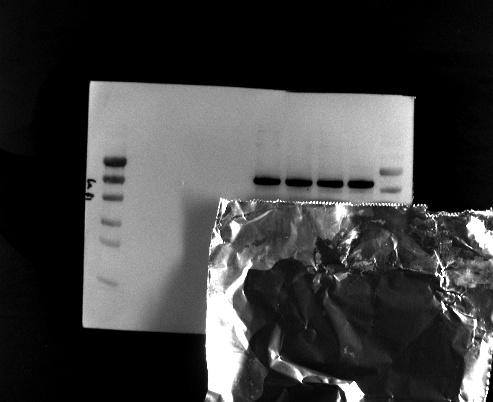


β-tubulin ALYREF







SRSF3


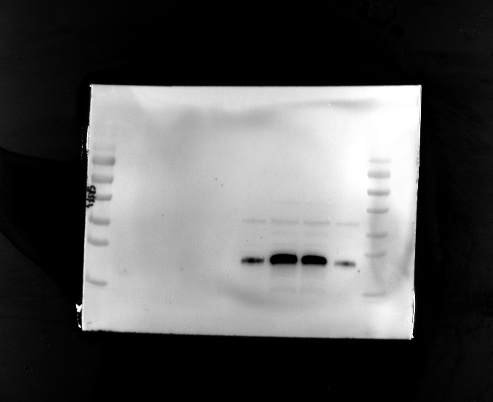


Fig. 8g

XPO5 Lamin B1




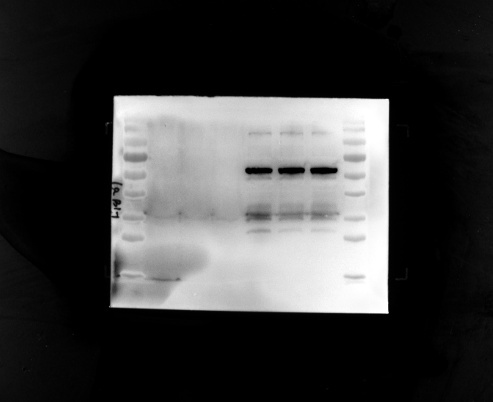


β-tubulin ALYREF







SRSF3





Fig. 6b

COL3A COL1A


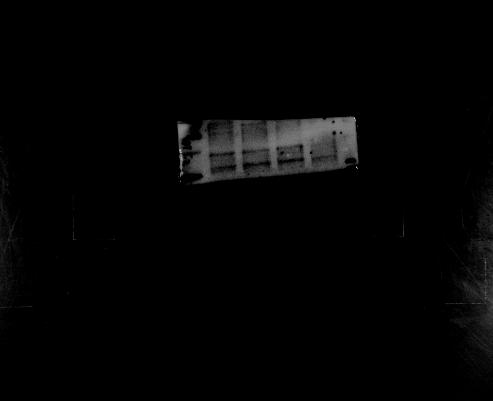

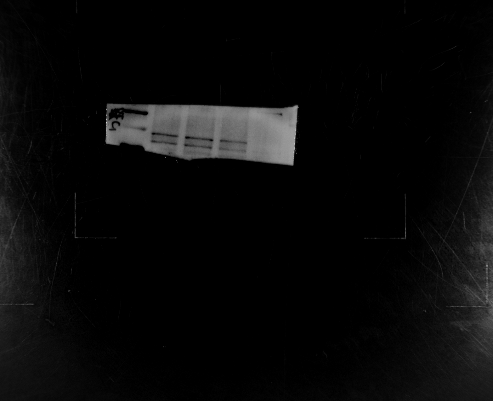


PICALM VIM


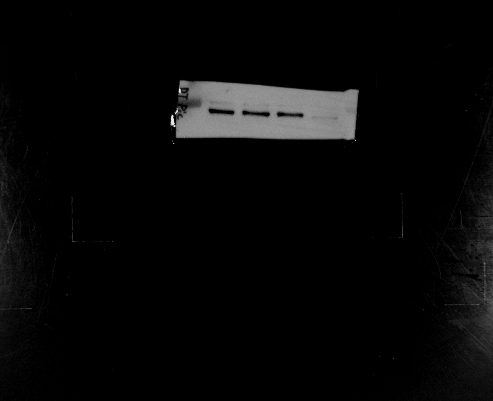

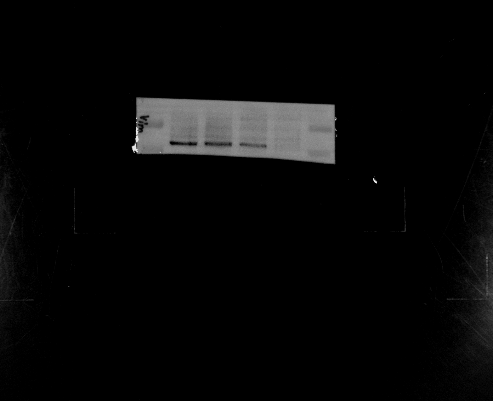


α-SMA GAPDH


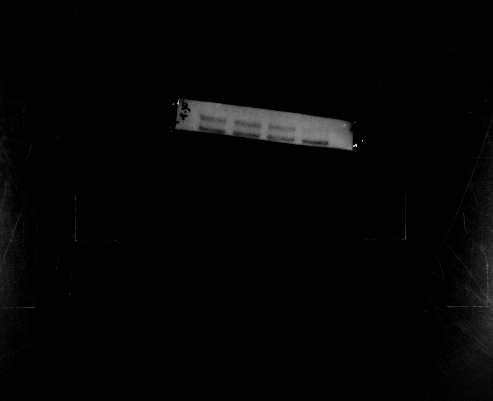

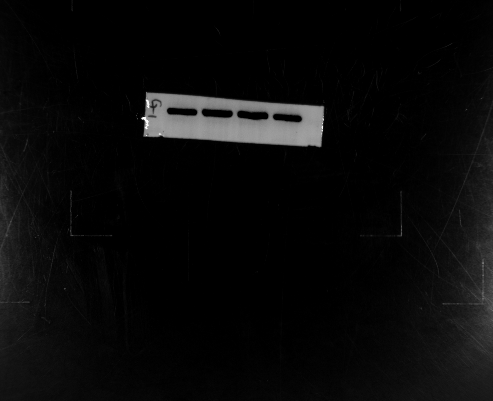


Fig. 6c

COL3A COL1A







FAP PICALM







VIM α-SMA




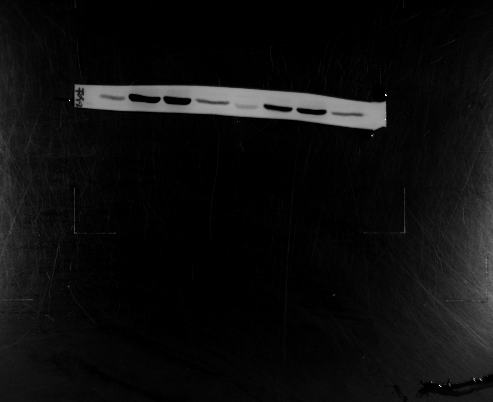


GAPDH





COL3A COL1A







FAP PICALM




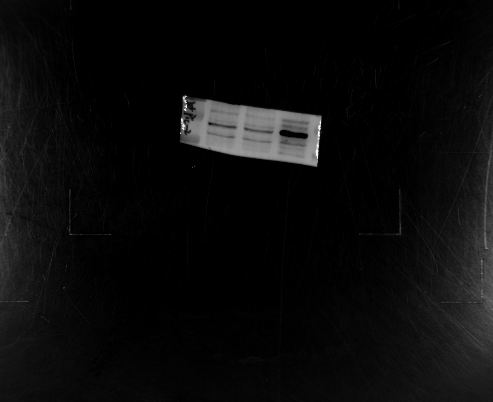


VIM α-SMA


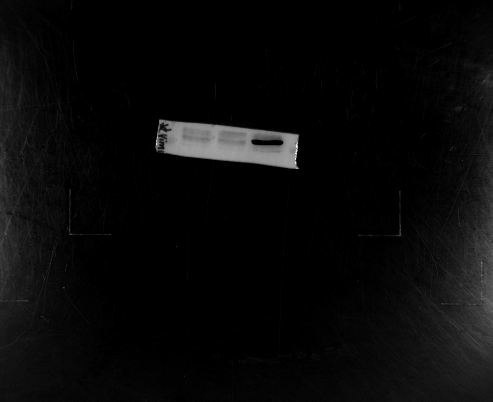

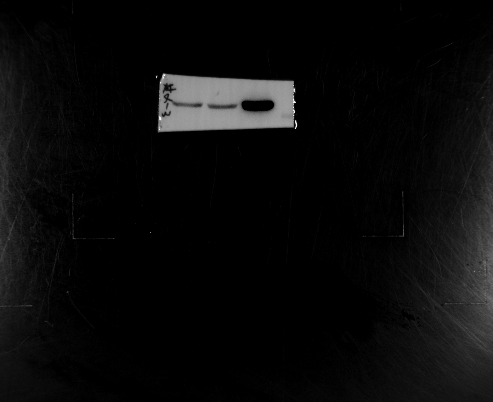


GAPDH





Fig. 7b

Upper left PICALM GAPDH







Upper right PICALM GAPDH







Lower left PICALM GAPDH







Lower right PICALM GAPDH







Fig. 7e

COL3A COL1A







FAP PICALM







VIM α-SMA







GAPDH





Fig. 7f

Left COL3A COL1A







YTHDC1 FAP


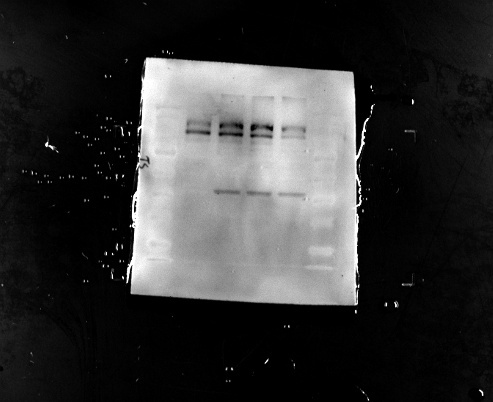




PICALM VIM




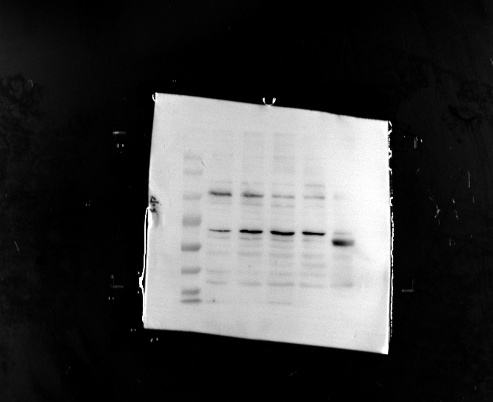


α-SMA GAPDH







Middle COL3A COL1A


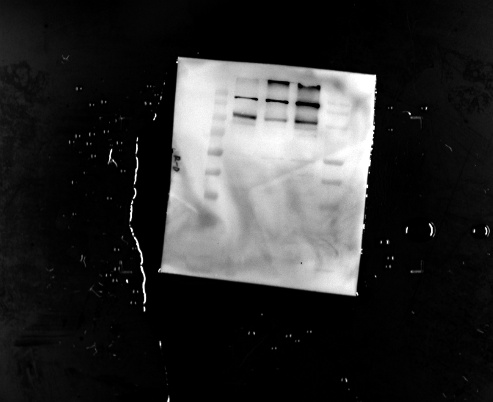


FAP PICALM

VIM α-SMA

GAPDH

Right COL3A COL1A

YTHDC1 FAP

PICALM VIM

α-SMA GAPDH

Fig. 7g

COL3A COL1A

FAP PICALM

VIM α-SMA

GAPDH

Fig. 7h

COL3A COL1A

FAP PICALM

VIM α-SMA

GAPDH

Fig. 8g

COL3A COL1A

FAP VIM

α-SMA GAPDH

COL3A COL1A

FAP VIM

α-SMA GAPDH

Supplementary Fig. 1d

Supplementary Fig. 1j

α-SMA α-SMA

α-SMA

Supplementary Fig. 2d

COL1A YTHDC1

FAP VIM

α-SMA GAPDH

Supplementary Fig. 2e

H3K9la GAPDH
